# Supplementary material for: Stereotyping in the digital age: Male language is “ingenious”, female language is “beautiful” – and popular
Source: PLoS One. 2020 Dec 16;15(12):e0243637. doi: 10.1371/journal.pone.0243637 (PMC7743969; doi:10.1371/journal.pone.0243637)
Supplement: S1 File — (DOCX) [file pone.0243637.s001.docx]

**Stereotyping in the digital age: Male language is “ingenious”, female language is “beautiful” – and popular**

Tabea Meier^1,2^*, Ryan L. Boyd^3^, Matthias R. Mehl^4^, Anne Milek^5^, James W. Pennebaker^6^, Mike Martin^1,2,7^, Markus Wolf^1^, and Andrea B. Horn^1,2^*

^1^ Department of Psychology, University of Zurich, Zurich, Switzerland

^2^University Research Priority Program “Dynamics of Healthy Aging”, University of Zurich, Zurich, Switzerland

^3^ Department of Psychology, Lancaster University, Lancaster, Lancashire, United Kingdom

^4^ Department of Psychology, The University of Arizona, Tucson, Arizona, United States of America

^5^ Department of Psychology, University of Münster, Muenster, North Rhine-Westphalia, Germany

^6^ Department of Psychology, The University of Texas, Austin, Texas, United States of America

^7^Collegium Helveticum, ETH Zurich, Zurich, Switzerland

Table of Contents

[Descriptive Statistics (Talk Ratings) **(Tables A-B)** - 3 -](#_Toc54625431)

[Quantity of Talk Impact, Main Results Including All Model Parameters: Number of Views By Gender and Gender-Linked Language (RQ1) **(Table C)** - 6 -](#_Toc54625432)

[Quantity of Talk Impact, Additional Analysis: Number of Talk Ratings by Gender and Gender-Linked Language (RQ1) **(Table D, Figure A)** - 7 -](#_Toc54625433)

[Quantity of Talk Impact, Main Results Including All Model Parameters: Number of Views by Age and Age-Linked Language (RQ2) **(Table E)** - 9 -](#_Toc54625434)

[Quantity of Talk Impact, Additional Analysis: Number of Talk Ratings by Age and Age-Linked Language (RQ2) **(Table F)** - 10 -](#_Toc54625435)

[Quality of Talk Impact: Main Results of Positive and Negative Rating Types by Gender and Gender-Linked Language (RQ1) **(Tables G-H)** - 11 -](#_Toc54625436)

[Quality of Talk Impact, Average Marginal Effects: Positive and Negative Rating Types by Gender and Gender-Linked Language (RQ1) **(Tables I-J)** - 17 -](#_Toc54625437)

[Quality of Talk Impact: Main Results of Positive and Negative Rating Types by Age and Age-Linked Language (RQ2) **(Tables K-L, Figures B-C)** - 22 -](#_Toc54625438)

[Quality of Talk Impact, Average Marginal Effects: Positive and Negative Rating Types by Age and Age-Linked Language (RQ2) **(Tables M-N)** - 26 -](#_Toc54625439)

[Quantity of Talk Impact, Additional Analysis During Peer-Review **(Tables O-P)** - 32 -](#_Toc54625440)

[References - 34 -](#_Toc54625441)

Descriptive Statistics (Talk Ratings) (Tables A-B)

| **Table A**  *Summary Information for TED Talks’ Absolute Number of Different Talk Ratings* | | | | | | |
| --- | --- | --- | --- | --- | --- | --- |
|  | Male speakers *N* = 747 | | | Female speakers *N* = 348 | | |
| Total Speakers  *N* = 1,095 | Total male  *N* = 747 | Academics  *N* = 158 | Non-academics  *N* = 589 | Total female  *N* = 348 | Academics  *N* = 62 | Non-academics  *N* = 286 |
| **Total number of ratings** ***M (SD)*** | | | | | | |
| 2,990.99 (5,598.32) | 2,942.60 (5,372.40) | 2,725.77 (3,431.83) | 3,000.76 (5,784.26) | 3,094.88 (6,061.69) | 5,225.16 (12,059.58) | 2,633.07 (3,522.56) |
| **Total positive ratings *M (SD)*** | | | | | | |
| 2,742.70 (5,425.15) | 2,696.97 (5,208.75) | 2,473.88 (3,312.08) | 2,756.81 (5,610.30) | 2,840.86 (5,869.42) | 4,904.44 (11,734.24) | 2,393.51 (3,368.42) |
| **Inspiring** ***M (SD)*** | | | | | | |
| 682.73 (1,814.61) | 648.53 (1,722.46) | 443.12 (948.31) | 703.63 (1,873.39) | 756.15 (1,998.78) | 1,293.13 (4,092.08) | 639.74 (1,097.20) |
| **Beautiful** ***M (SD)*** | | | | | | |
| 216.84 (538.42) | 189.76 (477.10) | 123.63 (398.14) | 207.50 (494.95) | 274.96 (647.80) | 341.92 (1,137.83) | 260.45 (482.34) |
| **Ingenious** ***M (SD)*** | | | | | | |
| 168.03 (350.05) | 191.57 (401.18) | 192.49 (289.10) | 191.32 (426.48) | 117.50 (191.17) | 174.35 (274.64) | 105.17 (165.81) |
| **Courageous** ***M (SD)*** | | | | | | |
| 219.38 (569.35) | 167.69 (345.54) | 119.54 (387.48) | 180.60 (332.56) | 330.32 (864.42) | 390.23 (1,400.48) | 317.34 (699.31) |
| **Jaw-dropping** ***M (SD)*** | | | | | | |
| 167.18 (494.10) | 182.95 (532.31) | 197.06 (631.64) | 179.17 (502.89) | 133.31 (398.56) | 213.66 (573.45) | 115.90 (348.30) |
| **Fascinating *M (SD)*** | | | | | | |
| 389.79 (744.13) | 402.07 (703.06) | 487.03 (667.16) | 379.28 (711.20) | 363.43 (825.89) | 826.77 (1,711.34) | 262.99 (383.15) |

| **Table A (continued)** | | | | | | |
| --- | --- | --- | --- | --- | --- | --- |
|  | Male speakers *N* = 747 | | | Female speakers *N* = 348 | | |
| Total Speakers  *N* = 1,095 | Total male  *N* = 747 | Academics  *N* = 158 | Non-academics  *N* = 589 | Total female  *N* = 348 | Academics  *N* = 62 | Non-academics  *N* = 286 |
| **Informative** ***M (SD)*** | | | | | | |
| 422.20 (715.63) | 414.02 (644.93) | 519.58 (557.80) | 385.71 (663.95) | 439.74 (848.50) | 992.74 (1,610.08) | 319.86 (490.68) |
| **Funny** ***M (SD)*** | | | | | | |
| 196.53 (852.50) | 209.80 (974.37) | 114.94 (281.65) | 235.25 (1,086.40) | 168.05 (499.00) | 206.89 (725.03) | 159.63 (436.19) |
| **Persuasive** ***M (SD)*** | | | | | | |
| 280.03 (640.12) | 290.58 (684.57) | 276.49 (385.84) | 294.35 (744.81) | 257.39 (532.53) | 464.74 (1,004.01) | 212.44 (343.71) |
| **Total negative ratings** ***M (SD)*** | | | | | | |
| 160.83 (239.60) | 159.99 (214.49) | 167.60 (172.62) | 157.95 (224.49) | 162.62 (286.49) | 186.52 (205.05) | 157.44 (301.30) |
| **Obnoxious** ***M (SD)*** | | | | | | |
| 31.80 (70.84) | 30.12 (66.25) | 25.74 (45.30) | 31.30 (70.81) | 35.40 (79.80) | 33.65 (38.48) | 35.78 (86.23) |
| **Longwinded** ***M (SD)*** | | | | | | |
| 41.59 (49.68) | 43.11 (50.03) | 46.66 (44.13) | 42.15 (51.49) | 38.35 (48.83) | 53.11 (67.05) | 35.15 (43.39) |
| **Unconvincing** ***M (SD)*** | | | | | | |
| 61.78 (115.83) | 60.65 (94.33) | 64.59 (80.84) | 59.59 (97.67) | 64.20 (152.19) | 68.15 (79.07) | 63.35 (163.88) |
| **Confusing *M (SD)*** | | | | | | |
| 25.65 (39.31) | 26.11 (38.33) | 30.61 (32.71) | 24.91 (39.64) | 24.66 (41.38) | 31.61 (39.21) | 23.16 (41.75) |
| **OK** ***M (SD)*** | | | | | | |
| 87.47 (106.11) | 85.64 (97.79) | 84.29 (69.81) | 86.00 (104.07) | 91.40 (122.13) | 134.21 (185.67) | 82.12 (101.47) |
| *Note. M* = Mean, *SD* = Standard deviation. Rating types are in bold face. Total positive ratings = aggregated score of all positive ratings, total negative ratings = aggregated score of all negative ratings, the rating “ok” was not considered in the aggregated ratings. | | | | | | |

| **Table B**  *Intercorrelations of TED Talk Ratings* | | | | | | | | | | | | | | | | | | |
| --- | --- | --- | --- | --- | --- | --- | --- | --- | --- | --- | --- | --- | --- | --- | --- | --- | --- | --- |
| Ratings (in %) | *M* | *SD* | (1) | (2) | (3) | (4) | (5) | (6) | (7) | (8) | (9) | (10) | (11) | (12) | (13) | (14) | (15) | (16) |
| (1) Positive | 87.79 | 10.41 | 1.00 |  |  |  |  |  |  |  |  |  |  |  |  |  |  |  |
| (2) Inspiring | 19.35 | 10.63 | .36^**^ | 1.00 |  |  |  |  |  |  |  |  |  |  |  |  |  |  |
| (3) Beautiful | 7.18 | 7.60 | .16^**^ | .28^**^ | 1.00 |  |  |  |  |  |  |  |  |  |  |  |  |  |
| (4) Ingenious | 6.26 | 5.93 | .10^**^ | -.18^**^ | -.13^**^ | 1.00 |  |  |  |  |  |  |  |  |  |  |  |  |
| (5) Courageous | 7.06 | 7.80 | .23^**^ | .38^**^ | .08^**^ | -.37^**^ | 1.00 |  |  |  |  |  |  |  |  |  |  |  |
| (6) Jaw-dropping | 4.70 | 5.38 | .25^**^ | -.21^**^ | .00 | .24^**^ | -.04 | 1.00 |  |  |  |  |  |  |  |  |  |  |
| (7) Fascinating | 13.07 | 7.38 | .16^**^ | -.43^**^ | -.04 | .31^**^ | -.51^**^ | .34^**^ | 1.00 |  |  |  |  |  |  |  |  |  |
| (8) Informative | 16.15 | 9.98 | .00 | -.47^**^ | -.49^**^ | -.14^**^ | -.25^**^ | -.16^**^ | .23^**^ | 1.00 |  |  |  |  |  |  |  |  |
| (9) Funny | 5.04 | 8.77 | 0.00 | -.23^**^ | -.09^**^ | .06 | -.19^**^ | -.11^**^ | -.04 | -.20^**^ | 1.00 |  |  |  |  |  |  |  |
| (10) Persuasive | 8.97 | 6.90 | 0.06 | .03 | -.41^**^ | -.25^**^ | .06 | -.22^**^ | -.36^**^ | .35^**^ | -.24^**^ | 1.00 |  |  |  |  |  |  |
| (11) Negative | 7.95 | 8.33 | -.97^**^ | -.33^**^ | -.16^**^ | -.10^**^ | -.19^**^ | -.21^**^ | -.18^**^ | -.05 | -.02 | -.03 | 1.00 |  |  |  |  |  |
| (12) Obnoxious | 1.45 | 2.17 | -.70^**^ | -.22^**^ | -.09^**^ | -.11^**^ | -.07^*^ | -.13^**^ | -.20^**^ | -.13^**^ | .08^*^ | -.05 | .76^**^ | 1.00 |  |  |  |  |
| (13) Confusing | 1.26 | 1.68 | -.70^**^ | -.32^**^ | -.10^**^ | .00 | -.20^**^ | -.13^**^ | -.01 | -.03 | .03 | -.13^**^ | .72^**^ | .38^**^ | 1.00 |  |  |  |
| (14) Longwinded | 2.24 | 2.76 | -.77^**^ | -.22^**^ | -.08^**^ | -.09^**^ | -.21^**^ | -.18^**^ | -.10^**^ | -.05 | -.02 | -.08^*^ | .76^**^ | .40^**^ | .55^**^ | 1.00 |  |  |
| (15) Unconvincing | 3.00 | 3.83 | -.85^**^ | -.30^**^ | -.20^**^ | -.09^**^ | -.13^**^ | -.19^**^ | -.20^**^ | .01 | -.08^**^ | .08^**^ | .88^**^ | .64^**^ | .51^**^ | .46^**^ | 1.00 |  |
| (16) OK | 4.26 | 3.15 | -.74^**^ | -.33^**^ | -.09^**^ | -.05 | -.28^**^ | -.28^**^ | -.06 | .12^**^ | .06^*^ | -.12^**^ | .55^**^ | .31^**^ | .43^**^ | .52^**^ | .46^**^ | 1.00 |
| *Note.* *N* = 1,095, *M* = Mean, *SD* = Standard deviation, Positive ratings = aggregated score of all positive ratings, negative ratings = aggregated score of all negative ratings, the rating “ok” was not considered in the aggregated ratings.  *p<0.05; **p<0.01; ***; *p*<0.001 | | | | | | | | | | | | | | | | | | |

Quantity of Talk Impact, Main Results Including All Model Parameters: Number of Views by Gender and Gender-Linked Language (RQ1) (Table C)

| **Table C**  *Research Question 1, TED Talk Impact (Number of Views) by Gender-Linked Language Style: Quantile Regression Results* | | | | | | | | | | | | | | | |
| --- | --- | --- | --- | --- | --- | --- | --- | --- | --- | --- | --- | --- | --- | --- | --- |
|  | Quantile | | | | | | | | | | | | | | |
|  | 10% | | | 25% | | | 50% | | | 75% | | | 90% | | |
|  | *B (SE)* | *t* | *p* | *B (SE)* | *t* | *p* | *B (SE)* | *t* | *p* | *B (SE)* | *t* | *p* | *B (SE)* | *t* | *p* |
| (Intercept) | 888,357.81  (57,021.13) | 15.58 | <.001^***^ | 1,137,396.39 (58,591.57) | 19.41 | <.001^***^ | 1,565,480.61 (108,358.92) | 14.45 | <.001^***^ | 2,199,713.64 (227,287.60) | 9.68 | <.001^***^ | 3,910,384.72 (809,021.83) | 4.83 | <.001^***^ |
| Academic status (academic) | 49,127.41 (26,913.73) | 1.83 | .068 | 111,819.78 (39,362.06) | 2.84 | .005^**^ | 97,070.73 (60,415.43) | 1.61 | .108 | 27,909.05 (140,346.24) | 0.20 | .842 | 878,549.93 (496,875.45) | 1.77 | .077 |
| Speaker's gender (male) | 16,287.39 (41,263.50) | 0.39 | .693 | 62,826.18 (31,463.13) | 2.00 | .046^*^ | 133,177.69 (57,113.11) | 2.33 | .020^*^ | 148,187.14 (150,013.54) | 0.99 | .323 | 1,195,798.39 (480,935.72) | 2.49 | .013^*^ |
| Time online | -117.49 (10.09) | -11.64 | <.001^***^ | -130.53  (12.23) | -10.67 | <.001^***^ | -101.28  (21.18) | -4.78 | <.001^***^ | -39.48  (44.70) | -0.88 | .377 | 77.11  (150.28) | 0.51 | .608 |
| Speaker's age | 37.87 (899.95) | 0.04 | .966 | -970.77  (1,175.77) | -0.83 | .409 | -4,660.02  (2,188.93) | -2.13 | .033^*^ | -6,804.04 (3,763.67) | -1.81 | .071 | -27,647.71 (13,245.84) | -2.09 | .037^*^ |
| Female language | 19,172.86 (16,090.30) | 1.19 | .234 | 46,571.72 (19,242.70) | 2.42 | .016^*^ | 123,285.13 (41,787.77) | 2.95 | .003^**^ | 286,225.26 (103,840.89) | 2.76 | .006^**^ | 723,286.42 (343,429.37) | 2.11 | .035^*^ |
| Speaker's gender × female language | -22,246.79 (22,266.04) | -1.00 | .318 | -21,385.99 (31,540.78) | -0.68 | .498 | -98,236.99 (56,741.29) | -1.73 | .084 | -94,118.19 (116,599.37) | -0.81 | .420 | 308,833.73 (468,603.13) | 0.66 | .510 |
| *Note.* Gender-linked language (“female language”) was z-standardized prior to inclusion in model. Higher values refer to a more female-typical style. Degrees of freedom: 1,095 total; 1,088 residual.  *p<0.05; **p<0.01; ***p<0.001 | | | | | | | | | | | | | | | |

Quantity of Talk Impact, Additional Analysis: Number of Talk Ratings by Gender and Gender-Linked Language (RQ1) (Table D, Figure A)

| **Table D**  *Additional Analysis: Number of Talk Ratings by Gender-Linked Language Style: Quantile Regression Results* | | | | | | | | | | | | | | | |
| --- | --- | --- | --- | --- | --- | --- | --- | --- | --- | --- | --- | --- | --- | --- | --- |
|  | Quantile | | | | | | | | | | | | | | |
|  | 10% | | | 25% | | | 50% | | | 75% | | | 90% | | |
|  | *B (SE)* | *t* | *p* | *B (SE)* | *t* | *p* | *B (SE)* | *t* | *p* | *B (SE)* | *t* | *p* | *B (SE)* | *t* | *p* |
| (Intercept) | 735.92 (85.51) | 8.61 | <.001^***^ | 1,127.39 (111.62) | 10.10 | <.001^***^ | 1,518.58 (190.37) | 7.98 | <.001^***^ | 2,493.94 (477.71) | 5.22 | <.001^***^ | 6,011.59 (1,310.32) | 4.59 | <.001^***^ |
| Academic status (academic) | 89.16 (67.85) | 1.31 | .189 | 177.41 (83.98) | 2.11 | .035 | 194.82 (138.84) | 1.40 | .161 | 90.73 (270.16) | 0.34 | .737 | 746.50 (872.81) | 0.86 | .393 |
| Speaker's gender (male) | -12.96 (46.27) | -0.28 | .780 | 83.50 (62.78) | 1.33 | .184 | 121.95 (108.98) | 1.12 | .263 | 324.65 (268.50) | 1.21 | .227 | -76.46 (831.81) | -0.09 | .927 |
| Time online | 0.00 (0.02) | -0.31 | .758 | -0.01 (0.02) | -0.58 | .564 | 0.07 (0.04) | 1.81 | .070 | 0.31 (0.10) | 2.93 | .003^**^ | 0.56 (0.26) | 2.21 | .028^*^ |
| Speaker's age | -1.84 (1.74) | -1.06 | .290 | -3.89 (2.27) | -1.71 | .087 | -2.81 (3.77) | -0.75 | .456 | -9.86 (9.72) | -1.01 | .311 | -33.47 (22.48) | -1.49 | .137 |
| Female language | 14.62 (30.62) | 0.48 | .633 | 81.44 (42.32) | 1.92 | .055 | 158.89 (75.69) | 2.10 | .036^*^ | 372.35 (190.04) | 1.96 | .050 | 1,508.47 (621.55) | 2.43 | .015^*^ |
| Speaker's gender × female language | -61.09 (45.38) | -1.35 | .179 | -62.78 (58.64) | -1.07 | .285 | -16.16 (101.45) | -0.16 | .873 | -124.09 (254.99) | -0.49 | .627 | -306.96 (729.88) | -0.42 | .674 |
| *Note.* Gender-linked language (“female language”) was z-standardized prior to inclusion in model, higher values refer to a more female language style. Degrees of freedom: 1095 total; 1088 residual.  *p<0.05; **p<0.01; ***p<0.001 | | | | | | | | | | | | | | | |

A more female-typical language style was linked to more talk ratings overall, but this relationship only held for the 50% and 90% quantiles of talk ratings. A one unit increase in female language style was linked to 158.89 (75.69) more talk ratings at the 50 % quantile, and 1,508.47 (621.55) at the 90 % quantile, respectively (see Table D and Figure A). Since the instruction to rate talks did not appear until the very end of each video, the larger number of overall ratings most likely suggests that talks given in female language were watched more often toward the end compared to talks given in male language. This implies that viewers enjoyed watching talks more when they where given in a female language style, regardless of the speaker’s presented gender.

**Figure A**

*Overall Number of Talk Ratings by Female-Typical Language Style*


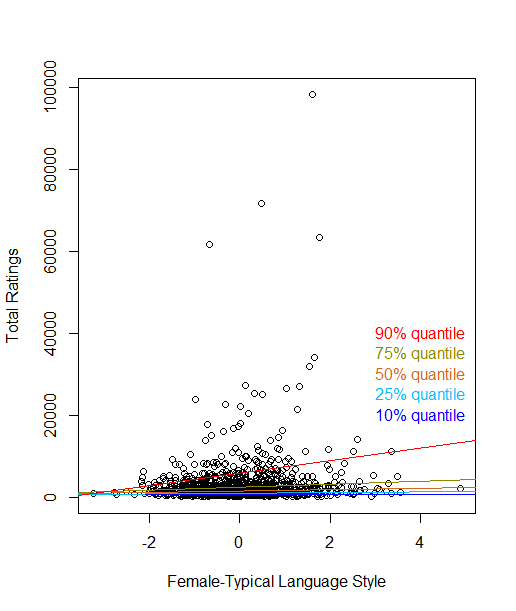


Note. Results from quantile regressions. Fitted regression lines separately for the 10%, 25%, 50%, 75%, and 90% quantiles of the total number of ratings indicate more pronounced positive associations between female language style and number of ratings among the often rated talks. Depicted are the effects of female-typical language style while accounting for control variables (speaker’s gender, age, academic status, time online; see Table D)

Quantity of Talk Impact, Main Results Including All Model Parameters: Number of Views by Age and Age-Linked Language (RQ2) (Table E)

| **Table E**  *Research Question 2, TED Talk Impact (Number of Views) by Age-Linked Language Style: Quantile Regression Results* | | | | | | | | | | | | | | | |
| --- | --- | --- | --- | --- | --- | --- | --- | --- | --- | --- | --- | --- | --- | --- | --- |
|  | Quantile | | | | | | | | | | | | | | |
|  | 10% | | | 25% | | | 50% | | | 75% | | | 90% | | |
|  | *B (SE)* | *t* | *p* | *B (SE)* | *t* | *p* | *B (SE)* | *t* | *p* | *B (SE)* | *t* | *p* | *B (SE)* | *t* | *p* |
| (Intercept) | 920,970.85 (44,651.75) | 20.63 | <.001^***^ | 1,103,188.86 (45,805.66) | 24.08 | <.001^***^ | 1,387,264.38 (82,568.33) | 16.80 | <.001^***^ | 2,132,865.51 (193,056.89) | 11.05 | <.001^***^ | 3,363,272.69 (621,551.76) | 5.41 | <.001^***^ |
| Academic status (academic) | 38,782.42 (21,076.32) | 1.84 | .066 | 105,005.72 (39,910.33) | 2.63 | .009^**^ | 110,082.81 (57,725.92) | 1.91 | .057 | -117,422.71 (166,152.59) | -0.71 | .480 | 729,646.78 (854,229.95) | 0.85 | .393 |
| Speaker's gender (male) | -10,762.64 (44,568.70) | -0.24 | .809 | 802.45  (47,294.57) | 0.02 | .986 | 44,843.81 (81,793.11) | 0.55 | .584 | -172,540.53 (204,976.24) | -0.84 | .400 | -436,451.71 (699,170.49) | -0.62 | .533 |
| Time online | -116.19  (10.19) | -11.40 | <.001^***^ | -129.24  (12.35) | -10.47 | <.001^***^ | -94.03  (20.93) | -4.49 | <.001^***^ | -40.97  (50.73) | -0.81 | .420 | 148.44  (140.83) | 1.05 | .292 |
| Age-linked language style | -793.77  (6,595.38) | -0.12 | .904 | 445.30  (3,919.06) | 0.11 | .910 | 802.68  (10,422.23) | 0.08 | .939 | -32,541.56 (28,286.41) | -1.15 | .250 | -71,557.88 (113,843.21) | -0.63 | .530 |
| Speaker’s age | 148.61  (1,953.83) | 0.08 | .939 | -163.90  (1,448.09) | -0.11 | .910 | -1,577.51  (3,456.80) | -0.46 | .648 | -1,264.05  (9,986.94) | -0.13 | .899 | -24,352.47 (33,175.23) | -0.73 | .463 |
| Age-linked language style  (squared) | -418.21  (438.60) | -0.95 | .341 | -284.27  (497.30) | -0.57 | .568 | -631.81  (991.13) | -0.64 | .524 | 1,002.22  (2,779.49) | 0.36 | .718 | -1,505.34  (15,034.09) | -0.10 | .920 |
| Speaker’s age (squared) | 126.46  (110.81) | 1.14 | .254 | 80.53  (100.09) | 0.80 | .421 | 18.53  (129.28) | 0.14 | .886 | -505.88  (540.19) | -0.94 | .349 | 397.47  (1,917.36) | 0.21 | .836 |
| speaker's gender (male)× Speaker’s age | -771.10  (2,788.33) | -0.28 | .782 | -3,251.76  (2,423.08) | -1.34 | .180 | -4,476.72  (4,189.72) | -1.07 | .286 | -7,246.11  (14,468.22) | -0.50 | .617 | -38,285.25 (49,855.85) | -0.77 | .443 |
| speaker's gender (male)× Speaker’s age(squared) | -56.18  (160.10) | -0.35 | .726 | 223.70  (130.47) | 1.71 | .087 | 71.92  (158.27) | 0.45 | .650 | 777.43  (880.17) | 0.88 | .377 | 1,675.23  (2,266.63) | 0.74 | .460 |
| speaker's gender (male)× Age-linked language style | -1,353.74 (7,679.47) | -0.18 | .860 | -801.09  (6,050.66) | -0.13 | .895 | -6,530.08 (1,1464.20) | -0.57 | .569 | 22,095.07 (35,796.17) | 0.62 | .537 | 136,066.01 (124,485.46) | 1.09 | .275 |
| speaker's gender (male)× Age-linked language style  (squared)^2^ | 193.49  (680.85) | 0.28 | .776 | 543.86  (753.65) | 0.72 | .471 | 1,075.54  (1,035.35) | 1.04 | .299 | 34.48  (4,379.59) | 0.01 | .994 | 1,757.77  (15,309.47) | 0.11 | .909 |
| *Note.* Speaker’s age and age-linked language style were mean-centered prior to model inclusion. Degrees of freedom: 1095 total; 1083 residual  *p<0.05; **p<0.01; ***p<0.001 | | | | | | | | | | | | | | | |

Quantity of Talk Impact, Additional Analysis: Number of Talk Ratings by Age and Age-Linked Language (RQ2) (Table F)

| **Table F**  *Additional Analysis: Number of Talk Ratings by Age-Linked Language Style: Quantile Regression Results* | | | | | | | | | | | | | | | |
| --- | --- | --- | --- | --- | --- | --- | --- | --- | --- | --- | --- | --- | --- | --- | --- |
|  | Quantile | | | | | | | | | | | | | | |
|  | 10% | | | 25% | | | 50% | | | 75% | | | 90% | | |
|  | *B (SE)* | *t* | *p* | *B (SE)* | *t* | *p* | *B (SE)* | *t* | *p* | *B (SE)* | *t* | *p* | *B (SE)* | *t* | *p* |
| (Intercept) | 668.52 (54.63) | 12.24 | <.001^***^ | 1,007.63 (91.69) | 10.99 | <.001*** | 1,593.41 (146.60) | 10.87 | <.001^***^ | 2,039.42 (364.79) | 5.59 | <.001^***^ | 4,834.09 (1,070.22) | 4.52 | <.001^***^ |
| Academic status (academic) | 83.11 (64.67) | 1.29 | .199 | 164.28 (71.88) | 2.29 | .022^*^ | 159.46 (118.28) | 1.35 | .178 | 98.31 (256.88) | 0.38 | .702 | 573.19 (1,079.41) | 0.53 | .596 |
| Speaker's gender (male) | -51.20 (59.95) | -0.85 | .393 | -8.23 (93.55) | -0.09 | .930 | -160.46 (151.81) | -1.06 | .291 | 171.68 (392.75) | 0.44 | .662 | -1,263.16 (1,119.73) | -1.13 | .260 |
| Time online | 0.00 (0.01) | 0.17 | .864 | -0.01 (0.02) | -0.63 | .526 | 0.06 (0.04) | 1.54 | .124 | 0.33 (0.09) | 3.62 | <.001^***^ | 0.63 (0.19) | 3.35 | .001^**^ |
| Age-linked language style | 2.13 (9.35) | 0.23 | .820 | 1.91 (8.05) | 0.24 | .813 | -1.44 (16.60) | -0.09 | .931 | 21.35 (21.96) | 0.97 | .331 | -65.16 (209.34) | -0.31 | .756 |
| Speaker’s age | -0.45 (2.66) | -0.17 | .865 | 0.08 (5.92) | 0.01 | .989 | -3.85 (9.52) | -0.40 | .686 | 15.37 (17.27) | 0.89 | .374 | -22.38 (56.87) | -0.39 | .694 |
| Age-linked language style (squared) | -0.67 (1.18) | -0.57 | .570 | -1.37 (0.91) | -1.51 | .132 | -2.96 (2.28) | -1.30 | .194 | -3.52 (2.56) | -1.37 | .170 | -6.44 (30.01) | -0.21 | .830 |
| Speaker’s age (squared) | 0.01 (0.14) | 0.07 | .946 | 0.15 (0.34) | 0.42 | .672 | -0.02 (0.61) | -0.04 | .971 | 1.10 (0.68) | 1.62 | .106 | 0.83 (1.65) | 0.50 | .614 |
| speaker's gender (male)× Speaker’s age | -0.79 (3.97) | -0.20 | .843 | -8.15 (6.91) | -1.18 | .239 | -6.65 (10.53) | -0.63 | .528 | -38.67 (22.35) | -1.73 | .084 | -59.15 (70.95) | -0.83 | .405 |
| speaker's gender (male)× Speaker’s age (squared) | -0.01 (0.19) | -0.05 | .962 | 0.20 (0.39) | 0.50 | .614 | 0.40 (0.64) | 0.63 | .532 | -1.09 (1.02) | -1.07 | .286 | 0.57 (2.10) | 0.27 | .786 |
| speaker's gender (male)× Age-linked language style | -6.62 (11.42) | -0.58 | .562 | -7.96 (11.32) | -0.70 | .482 | 12.68 (22.43) | 0.57 | .572 | 7.66 (32.66) | 0.23 | .815 | 139.71 (212.95) | 0.66 | .512 |
| speaker's gender (male)× Age-linked language style (squared) | 1.03 (1.49) | 0.69 | .491 | 1.21 (1.47) | 0.83 | .408 | 3.85 (3.26) | 1.18 | .237 | 5.14 (3.48) | 1.47 | .141 | 5.35 (30.40) | 0.18 | .860 |
| *Note.* Speaker’s age and age-linked language style were mean-centered prior to model inclusion. Degrees of freedom: 1095 total; 1083 residual  *p<0.05; **p<0.01; ***p<0.001. | | | | | | | | | | | | | | | |

Quality of Talk Impact: Main Results of Positive and Negative Rating Types by Gender and Gender-Linked Language (RQ1) (Tables G-H)

Tables G and H provide a detailed overview on the main results regarding gender, gender-linked language style and positive and negative talk ratings, including all model parameters. Since there were cases of zero for some of the ratings (our dependent variables), we applied the transformation procedure recommended in beta regressions on all our dependent variables of talk ratings: (y × (*N* − 1) + 0.5) / *N*; where *N* is the sample size (Smithson & Verkuilen, 2006). This transformation adds a small constant to y, forcing the variables to be bounded within the interval [0,1] without taking the extreme value of 0. This procedure was applied on all beta regression models in research questions 1 and 2.

| **Table G**  *Research Question 1, Positive TED Talk Ratings by Gender and Gender-Linked Language Style: Beta Regression Results* | | | | | | | | | | |
| --- | --- | --- | --- | --- | --- | --- | --- | --- | --- | --- |
| Dependent variables | Intercept | Female language | Speaker's gender (male) | Speaker's gender× female language | Speaker's age | Academic status (academic) | Total ratings | Time online | *R*^2^ | Log Likelihood |
| Positive ratings | 2.26*** | 0.04 | 0.07 | 0.04 | -0.002 | -0.06 | 0.0001*** | -0.0002*** | 0.16 | 1,356.64 |
| 95% CI | (2.08, 2.43) | (-0.03, 0.11) | (-0.03, 0.17) | (-0.05, 0.13) | (-0.01, 0.001) | (-0.16, 0.05) | (0.0001, 0.0001) | (-0.0002, -0.0001) |  |  |
| Cohen’s *d* |  | 0.02 | 0.04 | 0.02 | 0.00 | -0.03 | 0.000 | 0.000 |  |  |
| *p*-value | <.001 | .276 | .150 | .378 | .255 | .298 | <.001 | <.001 |  |  |
| Inspiring | -1.32*** | -0.003 | -0.08 | 0.04 | 0.004** | -0.39*** | 0.0000*** | -0.0001*** | 0.11 | 1,030.66 |
| 95% CI | (-1.47, -1.16) | (-0.06, 0.06) | (-0.17, 0.003) | (-0.04, 0.12) | (0.001, 0.01) | (-0.49, -0.29) | (0.0000, 0.0000) | (-0.0001, -0.0001) |  |  |
| Cohen’s *d* |  | -0.002 | -0.05 | 0.02 | 0.00 | -0.22 | 0.00 | 0.00 |  |  |
| *p*-value | <.001 | .926 | .059 | .368 | .007 | <.001 | <.001 | <.001 |  |  |
| Beautiful | -2.14*** | 0.10** | -0.25*** | 0.07 | -0.001 | -0.33*** | 0.00 | -0.0001** | 0.13 | 1,831.98 |
| 95% CI | (-2.33, -1.94) | (0.03, 0.17) | (-0.36, -0.14) | (-0.02, 0.17) | (-0.005, 0.003) | (-0.46, -0.21) | (-0.0000, 0.0000) | (-0.0001, -0.0000) |  |  |
| Cohen’s *d* |  | 0.06 | -0.14 | 0.04 | -0.001 | -0.18 | 0.000 | 0.000 |  |  |
| *p*-value | <.001 | .005 | <.001 | .140 | .623 | <.001 | .615 | .002 |  |  |
| Ingenious | -2.60*** | -0.13** | 0.31*** | 0.09 | -0.01*** | 0.15** | 0.00 | 0.0000* | 0.10 | 1,993.41 |
| 95% CI | (-2.79,  -2.41) | (-0.20, -0.05) | (0.20, 0.41) | (-0.01, 0.18) | (-0.01, -0.01) | (0.04, 0.26) | (-0.0000, 0.0000) | (0.0000, 0.0001) |  |  |
| Cohen’s *d* |  | -0.07 | 0.17 | 0.05 | -0.005 | 0.08 | 0.000 | 0.000 |  |  |
| *p*-value | <.001 | .002 | <.001 | .085 | <.001 | .007 | .260 | .025 |  |  |

| **Table G (continued)** | | | | | | | | | | |
| --- | --- | --- | --- | --- | --- | --- | --- | --- | --- | --- |
| Dependent variables | Intercept | Female language | Speaker's gender (male) | Speaker's gender× female language | Speaker's age | Academic status (academic) | Total ratings | Time online | *R*^2^ | Log Likelihood |
| Courageous | -2.12*** | 0.10** | -0.29*** | -0.07 | -0.0002 | -0.45*** | 0.00 | -0.0001*** | 0.13 | 1,854.17 |
| 95% CI | (-2.32, -1.92) | (0.02, 0.17) | (-0.41, -0.18) | (-0.17, 0.03) | (-0.004, 0.004) | (-0.58, -0.32) | (-0.0000, 0.0000) | (-0.0001, -0.0000) |  |  |
| Cohen’s *d* |  | 0.05 | -0.16 | -0.04 | 0.000 | -0.25 | 0.000 | 0.000 |  |  |
| *p*-value | <.001 | .010 | <.001 | .158 | .924 | <.001 | .224 | <.001 |  |  |
| Jaw-dropping | -3.09*** | -0.07 | 0.11* | 0.02 | -0.004* | 0.06 | 0.0000* | 0.0001*** | 0.06 | 2,271.18 |
| 95% CI | (-3.28, -2.90) | (-0.14, 0.01) | (0.004, 0.21) | (-0.08, 0.11) | (-0.01, -0.001) | (-0.05, 0.17) | (0.0000, 0.0000) | (0.0000, 0.0001) |  |  |
| Cohen’s *d* |  | -0.04 | 0.06 | 0.01 | -0.002 | 0.03 | 0.000 | 0.000 |  |  |
| *p*-value | <.001 | .070 | .043 | .754 | .023 | .323 | .017 | <.001 |  |  |
| Fascinating | -2.03 | -0.06 | 0.15*** | 0.03 | 0.00 | 0.37*** | 0.00 | 0.0000* | 0.10 | 1,469.43 |
| 95% CI | (-2.18, -1.89) | (-0.12, -0.01) | (0.06, 0.23) | (-0.04, 0.11) | (-0.01, 0.0003) | (0.29, 0.45) | (-0.0000, 0.0000) | (0.0000, 0.0001) |  |  |
| Cohen’s *d* |  | -0.04 | 0.08 | 0.02 | -0.001 | 0.20 | 0.000 | 0.000 |  |  |
| *p*-value | <.001 | .031 | <.001 | .379 | .082 | <.001 | .882 | .024 |  |  |
| Informative | -1.61*** | -0.13*** | -0.06 | -0.04 | 0.001 | 0.59*** | -0.0000*** | -0.0001*** | 0.17 | 1,165.12 |
| 95% CI | (-1.77, -1.44) | (-0.20, -0.07) | (-0.15, 0.03) | (-0.13, 0.05) | (-0.002, 0.005) | (0.50, 0.69) | (-0.0000,  -0.0000) | (-0.0001, -0.0000) |  |  |
| Cohen’s *d* |  | -0.07 | -0.03 | -0.02 | 0.001 | 0.33 | 0.000 | 0.000 |  |  |
| *p*-value | <.001 | <.001 | .200 | .364 | .378 | <.001 | .001 | <.001 |  |  |

| **Table G (continued)** | | | | | | | | | | |
| --- | --- | --- | --- | --- | --- | --- | --- | --- | --- | --- |
| Dependent variables | Intercept | Female language | Speaker's gender (male) | Speaker's gender× female language | Speaker's age | Academic status (academic) | Total ratings | Time online | *R*^2^ | Log Likelihood |
| Funny | -3.24*** | 0.29*** | 0.26*** | -0.02 | -0.001 | -0.09 | 0.0000** | 0.0001*** | 0.13 | 2,329.53 |
| 95% CI | (-3.47, -3.00) | (0.21, 0.38) | (0.13, 0.39) | (-0.13, 0.09) | (-0.01, 0.003) | (-0.23, 0.04) | (0.0000, 0.0000) | (0.0000, 0.0001) |  |  |
| Cohen’s *d* |  | 0.16 | 0.15 | -0.01 | -0.001 | -0.05 | 0.000 | 0.000 |  |  |
| *p*-value | <.001 | <.001 | <.001 | .716 | .535 | .178 | .006 | <.001 |  |  |
| Persuasive | -2.57*** | -0.08* | -0.07 | -0.08 | 0.01*** | 0.14** | 0.0000* | -0.0001** | 0.07 | 1,634.65 |
| 95% CI | (-2.75, -2.39) | (-0.15, -0.01) | (-0.17, 0.03) | (-0.17, 0.01) | (0.004, 0.01) | (0.04, 0.25) | (0.0000, 0.0000) | (-0.0001, -0.0000) |  |  |
| Cohen’s *d* |  | -0.05 | -0.04 | -0.04 | 0.004 | 0.08 | 0.000 | 0.000 |  |  |
| *p*-value | <.001 | .021 | .195 | .090 | <.001 | .009 | .011 | .003 |  |  |
| *Note.* *N* = 1,095, CI = Confidence interval of estimates. Gender-linked language (“female language”) was z-standardized prior to inclusion in model, higher values on the score refer to a more female-typical language style. All ratings represent percentages of total ratings received. Positive ratings = aggregated score of all positive ratings.  *p<0.05; **p<0.01; ***p<0.001 | | | | | | | | | | |

| **Table H**  *Research Question 1, Negative TED Talk Ratings by Gender and Gender-Linked Language Style: Beta Regression Results* | | | | | | | | | | |
| --- | --- | --- | --- | --- | --- | --- | --- | --- | --- | --- |
|  | Intercept | Female language | speaker's gender (male) | speaker's gender × female language | speaker's age | academic status (academic) | total ratings | time online | *R*^2^ | Log Likelihood |
| Negative ratings | -2.89*** | -0.06 | -0.09 | -0.05 | 0.004* | 0.04 | -0.0001*** | 0.0002*** | 0.18 | 1,759.78 |
| 95% CI | (-3.08, -2.70) | (-0.13, 0.02) | (-0.20, 0.01) | (-0.14, 0.05) | (0.001, 0.01) | (-0.07, 0.15) | (-0.0001, -0.0000) | (0.0001, 0.0002) |  |  |
| Cohen’s *d* |  | -0.03 | -0.05 | -0.03 | 0.002 | 0.02 | 0.000 | 0.000 |  |  |
| *p*-value | <.001 | .136 | .085 | .344 | .024 | .481 | <.001 | <.001 |  |  |
| Obnoxious | -4.34*** | 0.002 | -0.11* | -0.07 | -0.001 | -0.08 | -0.0000*** | 0.0001*** | 0.10 | 3,542.16 |
| 95% CI | (-4.53, -4.14) | (-0.07, 0.08) | (-0.22, -0.01) | (-0.16, 0.03) | (-0.005, 0.003) | (-0.19, 0.04) | (-0.0000, -0.0000) | (0.0001, 0.0002) |  |  |
| Cohen’s *d* |  | 0.001 | -0.06 | -0.04 | -0.001 | -0.04 | 0.000 | 0.000 |  |  |
| *p*-value | <.001 | .955 | .037 | .184 | .599 | .188 | <.001 | <.001 |  |  |
| Longwinded | -4.57*** | -0.07* | -0.09 | 0.06 | 0.01*** | 0.07 | -0.0001*** | 0.0002*** | 0.22 | 3,169.45 |
| 95% CI | (-4.76, -4.39) | (-0.14, -0.0001) | (-0.19, 0.01) | (-0.03, 0.16) | (0.005, 0.01) | (-0.04, 0.17) | (-0.0001, -0.0000) | (0.0002, 0.0003) |  |  |
| Cohen’s *d* |  | -0.04 | -0.05 | 0.03 | 0.005 | 0.04 | 0.000 | 0.000 |  |  |
| *p*-value | <.001 | .050 | .086 | .186 | <.001 | .213 | <.001 | <.001 |  |  |
| Unconvincing | -3.63*** | -0.09* | -0.11* | -0.08 | 0.001 | 0.01 | -0.0000*** | 0.0001*** | 0.12 | 2,768.51 |
| 95% CI | (-3.83, -3.43) | (-0.17, -0.01) | (-0.22, -0.001) | (-0.18, 0.02) | (-0.002, 0.005) | (-0.11, 0.13) | (-0.0000, -0.0000) | (0.0001, 0.0001) |  |  |
| Cohen’s *d* |  | -0.05 | -0.06 | -0.04 | 0.001 | 0.01 | 0.000 | 0.000 |  |  |
| *p*-value | <.001 | .021 | .049 | .115 | .516 | .860 | <.001 | <.001 |  |  |

| **Table H (continued)** | | | | | | | | | | |
| --- | --- | --- | --- | --- | --- | --- | --- | --- | --- | --- |
|  | Intercept | Female language | speaker's gender (male) | speaker's gender × female language | speaker's age | academic status (academic) | total ratings | time online | *R*^2^ | Log Likelihood |
| Confusing | -4.65*** | -0.01 | 0.01 | -0.07 | 0.001 | 0.18** | -0.0000*** | 0.0001*** | 0.10 | 3,699.64 |
| 95% CI | (-4.84, -4.46) | (-0.08, 0.07) | (-0.10, 0.11) | (-0.16, 0.03) | (-0.003, 0.004) | (0.07, 0.28) | (-0.0000, -0.0000) | (0.0001, 0.0001) |  |  |
| Cohen’s *d* |  | -0.004 | 0.004 | -0.04 | 0.000 | 0.10 | 0.000 | 0.000 |  |  |
| *p*-value | <.001 | .853 | .881 | .174 | .631 | .002 | <.001 | <.001 |  |  |
| OK_1_ | -3.04*** | -0.01 | -0.04 | -0.01 | -0.003 | 0.09* | -0.0001*** | 0.0001*** | 0.10 | 2,561.40 |
| 95% CI | (-3.19, -2.89) | (-0.07, 0.04) | (-0.13, 0.04) | (-0.09, 0.06) | (-0.01, 0.0000) | (0.002, 0.18) | (-0.0001, -0.0001) | (0.0001, 0.0001) |  |  |
| Cohen’s *d* |  | -0.01 | -0.02 | -0.01 | -0.002 | 0.05 | 0.000 | 0.000 |  |  |
| *p*-value | <.001 | .642 | .314 | .724 | .055 | .046 | <.001 | <.001 |  |  |
| *Note.* *N* = 1,095, CI = Confidence interval of estimates. Gender-linked language (“female language”) was z-standardized prior to inclusion in model, higher values on the score refer to a more female language style. All ratings represent percentages of total ratings received. Negative ratings = aggregated score of all negative ratings.  _1_The rating “ok” was not part of the aggregated score of “negative ratings”.  *p<0.05; **p<0.01; ***p<0.001 | | | | | | | | | | |

Quality of Talk Impact, Average Marginal Effects: Positive and Negative Rating Types by Gender and Gender-Linked Language (RQ1) (Tables I-J)

Average marginal effects (AME) represent the average model coefficients. As an example, TED Talks, on average, received 5.04% “funny” ratings (Table 2). The AME of gender-linked language style (standardized femininity score) on “funny” was 0.014 (see Table I). A one standard deviation change towards a more female-typical language style thus corresponded to a 1.4% increase in “funny” ratings when the other model predictors were held constant. Similarly, presenting as a male (rather than female) speaker linked to a 2.1% decrease in “courageous” ratings.

| **Table I**  *Research Question 1, Positive TED Talk Ratings by Gender and Gender-Linked Language Style: Average Marginal Effects From Beta Regressions* | | | | | | |
| --- | --- | --- | --- | --- | --- | --- |
| **Dependent variable** | Predictor | AME | SE | Lower CI | Upper CI | *p* |
| **Positive ratings** |  |  |  |  |  |  |
|  | Academic status (academic) | -0.006 | 0.006 | -0.018 | 0.006 | .303 |
|  | Speaker's age | 0.000 | 0.000 | -0.001 | 0.000 | .254 |
|  | Speaker’s gender (male) | 0.008 | 0.006 | -0.003 | 0.018 | .175 |
|  | Time online | 0.000 | 0.000 | 0.000 | 0.000 | <.001 |
|  | Total ratings | 0.000 | 0.000 | 0.000 | 0.000 | <.001 |
|  | Female language | 0.007 | 0.003 | 0.002 | 0.012 | .005 |
| **Inspiring** |  |  |  |  |  |  |
|  | Academic status (academic) | -0.057 | 0.007 | -0.070 | -0.043 | <.001 |
|  | Speaker's age | 0.001 | 0.000 | 0.000 | 0.001 | .007 |
|  | Speaker’s gender (male) | -0.013 | 0.007 | -0.027 | 0.001 | .064 |
|  | Time online | 0.000 | 0.000 | 0.000 | 0.000 | <.001 |
|  | Total ratings | 0.000 | 0.000 | 0.000 | 0.000 | <.001 |
|  | Female language | 0.003 | 0.003 | -0.003 | 0.010 | .305 |
| **Beautiful** |  |  |  |  |  |  |
|  | Academic status (academic) | -0.021 | 0.004 | -0.028 | -0.014 | <.001 |
|  | Speaker's age | 0.000 | 0.000 | 0.000 | 0.000 | .623 |
|  | Speaker’s gender (male) | -0.017 | 0.004 | -0.025 | -0.009 | <.001 |
|  | Time online | 0.000 | 0.000 | 0.000 | 0.000 | .001 |
|  | Total ratings | 0.000 | 0.000 | 0.000 | 0.000 | .615 |
|  | Female language | 0.010 | 0.002 | 0.006 | 0.013 | <.001 |
| **Ingenious** |  |  |  |  |  |  |
|  | Academic status (academic) | 0.009 | 0.004 | 0.002 | 0.016 | .009 |
|  | Speaker's age | -0.001 | 0.000 | -0.001 | 0.000 | <.001 |
|  | Speaker’s gender (male) | 0.017 | 0.003 | 0.011 | 0.023 | <.001 |
|  | Time online | 0.000 | 0.000 | 0.000 | 0.000 | .025 |
|  | Total ratings | 0.000 | 0.000 | 0.000 | 0.000 | .260 |
|  | Female language | -0.004 | 0.002 | -0.007 | -0.001 | .014 |
| **Courageous** |  |  |  |  |  |  |
|  | Academic status (academic) | -0.026 | 0.003 | -0.033 | -0.020 | <.001 |
|  | Speaker's age | 0.000 | 0.000 | 0.000 | 0.000 | .923 |
|  | Speaker’s gender (male) | -0.021 | 0.004 | -0.028 | -0.013 | <.001 |
|  | Time online | 0.000 | 0.000 | 0.000 | 0.000 | <.001 |
|  | Total ratings | 0.000 | 0.000 | 0.000 | 0.000 | .224 |
|  | Female language | 0.003 | 0.002 | 0.000 | 0.007 | .047 |

| **Table I (continued)** | | | | | | |
| --- | --- | --- | --- | --- | --- | --- |
| **Dependent Variable** | Predictor | AME | SE | Lower CI | Upper CI | *p* |
| **Jaw-dropping** |  |  |  |  |  |  |
|  | Academic status (academic) | 0.003 | 0.003 | -0.003 | 0.008 | .330 |
|  | Speaker's age | 0.000 | 0.000 | 0.000 | 0.000 | .023 |
|  | Speaker’s gender (male) | 0.005 | 0.002 | 0.000 | 0.010 | .042 |
|  | Time online | 0.000 | 0.000 | 0.000 | 0.000 | <.001 |
|  | Total ratings | 0.000 | 0.000 | 0.000 | 0.000 | .017 |
|  | Female language | -0.003 | 0.001 | -0.005 | 0.000 | .022 |
| **Fascinating** |  |  |  |  |  |  |
|  | Academic status (academic) | 0.046 | 0.006 | 0.035 | 0.056 | <.001 |
|  | Speaker's age | 0.000 | 0.000 | -0.001 | 0.000 | .081 |
|  | Speaker’s gender (male) | 0.016 | 0.005 | 0.007 | 0.025 | .000 |
|  | Time online | 0.000 | 0.000 | 0.000 | 0.000 | .024 |
|  | Total ratings | 0.000 | 0.000 | 0.000 | 0.000 | .882 |
|  | Female language | -0.005 | 0.002 | -0.009 | 0.000 | .039 |
| **Informative** |  |  |  |  |  |  |
|  | Academic status (academic) | 0.088 | 0.008 | 0.073 | 0.104 | <.001 |
|  | Speaker's age | 0.000 | 0.000 | 0.000 | 0.001 | .377 |
|  | Speaker’s gender (male) | -0.008 | 0.006 | -0.020 | 0.005 | .243 |
|  | Time online | 0.000 | 0.000 | 0.000 | 0.000 | <.001 |
|  | Total ratings | 0.000 | 0.000 | 0.000 | 0.000 | .001 |
|  | Female language | -0.021 | 0.003 | -0.027 | -0.016 | <.001 |
| **Funny** |  |  |  |  |  |  |
|  | Academic status (academic) | -0.005 | 0.003 | -0.011 | 0.002 | .167 |
|  | Speaker's age | 0.000 | 0.000 | 0.000 | 0.000 | .535 |
|  | Speaker’s gender (male) | 0.012 | 0.003 | 0.007 | 0.018 | <.001 |
|  | Time online | 0.000 | 0.000 | 0.000 | 0.000 | <.001 |
|  | Total ratings | 0.000 | 0.000 | 0.000 | 0.000 | .006 |
|  | Female language | 0.014 | 0.002 | 0.011 | 0.017 | <.001 |
| **Persuasive** |  |  |  |  |  |  |
|  | Academic status (academic) | 0.012 | 0.005 | 0.003 | 0.021 | .011 |
|  | Speaker's age | 0.001 | 0.000 | 0.000 | 0.001 | <.001 |
|  | Speaker’s gender (male) | -0.005 | 0.004 | -0.013 | 0.004 | .259 |
|  | Time online | 0.000 | 0.000 | 0.000 | 0.000 | .002 |
|  | Total ratings | 0.000 | 0.000 | 0.000 | 0.000 | .010 |
|  | Female language | -0.011 | 0.002 | -0.015 | -0.007 | <.001 |
| *Note.* AME = Average marginal effects (type = response; computed with the R-package “margins”; Leeper, 2018), SE = standard error, CI = 95% Confidence Interval.  Gender-linked language (“female language”) was z-standardized prior to inclusion in model, higher values on the score refer to a more female-typical language style. All ratings represent percentages of total ratings received. Positive ratings = aggregated score of all positive ratings. | | | | | | |

| **Table J**  *Research Question 1, Negative TED Talk Ratings by Gender and Gender-Linked Language Style: Average Marginal Effects From Beta Regressions* | | | | | | |
| --- | --- | --- | --- | --- | --- | --- |
| **Dependent variable** | **Predictor** | **AME** | **SE** | **Lower CI** | **Upper CI** | ***p*** |
| **Negative ratings** |  |  |  |  |  |  |
|  | Academic status (academic) | 0.003 | 0.004 | -0.005 | 0.011 | .485 |
|  | Speaker's age | 0.000 | 0.000 | 0.000 | 0.001 | .023 |
|  | Speaker’s gender (male) | -0.007 | 0.004 | -0.015 | 0.002 | .111 |
|  | Time online | 0.000 | 0.000 | 0.000 | 0.000 | <.001 |
|  | Total ratings | 0.000 | 0.000 | 0.000 | 0.000 | <.001 |
|  | Female language | -0.007 | 0.002 | -0.010 | -0.003 | .001 |
| **Obnoxious:** |  |  |  |  |  |  |
|  | Academic status (academic) | -0.001 | 0.001 | -0.003 | 0.001 | .177 |
|  | Speaker's age | 0.000 | 0.000 | 0.000 | 0.000 | .598 |
|  | Speaker’s gender (male) | -0.002 | 0.001 | -0.003 | 0.000 | .047 |
|  | Time online | 0.000 | 0.000 | 0.000 | 0.000 | <.001 |
|  | Total ratings | 0.000 | 0.000 | 0.000 | 0.000 | <.001 |
|  | Female language | -0.001 | 0.000 | -0.001 | 0.000 | .096 |
| **Longwinde** |  |  |  |  |  |  |
|  | Academic status (academic) | 0.002 | 0.001 | -0.001 | 0.004 | .221 |
|  | Speaker's age | 0.000 | 0.000 | 0.000 | 0.000 | <.001 |
|  | Speaker’s gender (male) | -0.002 | 0.001 | -0.005 | 0.000 | .081 |
|  | Time online | 0.000 | 0.000 | 0.000 | 0.000 | <.001 |
|  | Total ratings | 0.000 | 0.000 | 0.000 | 0.000 | <.001 |
|  | Female language | -0.001 | 0.001 | -0.002 | 0.001 | .271 |
| **Unconvincing** |  |  |  |  |  |  |
|  | Academic status (academic) | 0.000 | 0.002 | -0.003 | 0.004 | .860 |
|  | Speaker's age | 0.000 | 0.000 | 0.000 | 0.000 | .516 |
|  | Speaker’s gender (male) | -0.003 | 0.002 | -0.006 | 0.000 | .087 |
|  | Time online | 0.000 | 0.000 | 0.000 | 0.000 | <.001 |
|  | Total ratings | 0.000 | 0.000 | 0.000 | 0.000 | <.001 |
|  | Female language | -0.004 | 0.001 | -0.006 | -0.003 | <.001 |

| **Table J (continued)** | | | | | | |
| --- | --- | --- | --- | --- | --- | --- |
| **Dependent variable** | **Predictor** | **AME** | **SE** | **Lower CI** | **Upper CI** | ***p*** |
| **Confusing** |  |  |  |  |  |  |
|  | Academic status (academic) | 0.002 | 0.001 | 0.001 | 0.004 | .003 |
|  | Speaker's age | 0.000 | 0.000 | 0.000 | 0.000 | .631 |
|  | Speaker’s gender (male) | 0.000 | 0.001 | -0.001 | 0.002 | .814 |
|  | Time online | 0.000 | 0.000 | 0.000 | 0.000 | <.001 |
|  | Total ratings | 0.000 | 0.000 | 0.000 | 0.000 | <.001 |
|  | Female language | -0.001 | 0.000 | -0.001 | 0.000 | .032 |
| **Ok_1_** |  |  |  |  |  |  |
|  | Academic status (academic) | 0.004 | 0.002 | 0.000 | 0.008 | .051 |
|  | Speaker's age | 0.000 | 0.000 | 0.000 | 0.000 | .054 |
|  | Speaker’s gender (male) | -0.002 | 0.002 | -0.005 | 0.002 | .328 |
|  | Time online | 0.000 | 0.000 | 0.000 | 0.000 | <.001 |
|  | Total ratings | 0.000 | 0.000 | 0.000 | 0.000 | <.001 |
|  | Female language | -0.001 | 0.001 | -0.003 | 0.001 | .248 |
| *Note.* AME = Average marginal effects (type = response; computed with the R-package “margins”; Leeper, 2018), SE = standard error, CI = 95% Confidence Interval.  Gender-linked language (“female language”) was z-standardized prior to inclusion in model, higher values on the score refer to a more female-typical language style. All ratings represent percentages of total ratings received. Negative ratings = aggregated score of all negative ratings.  _1_The rating “ok” was not part of the aggregated score of “negative ratings”. | | | | | | |

Quality of Talk Impact: Main Results of Positive and Negative Rating Types by Age and Age-Linked Language (RQ2) (Tables K-L, Figures B-C)

Tables K and L provide a detailed overview on the main results regarding age, age-linked language style and positive and negative talk ratings, including all model parameters.

| **Table K**  *Research Question 2, Positive TED Talk Ratings by Age and Age-Linked Language Style: Beta Regression Results* | | | | | | | | | | | | | | | |
| --- | --- | --- | --- | --- | --- | --- | --- | --- | --- | --- | --- | --- | --- | --- | --- |
| Dependent variables | Intercept | Age-linked language style | Speaker’s age | Age-linked language style (squared) | Speaker’s age (squared) | academic status (academic) | speaker's gender (male) | speaker's gender (male)× Age-linked language style | speaker's gender (male)× Age-linked language style (squared) | speaker's gender (male)× Speaker’s age | speaker's gender (male)× Speaker’s age (squared) | Total ratings | Time online | *R*^2^ | Log Likelihood |
| Positive ratings | 2.17*** | 0.001 | -0.001 | -0.0003 | 0.0001 | -0.07 | 0.07 | 0.0002 | 0.0004 | -0.001 | -0.0003 | 0.0001*** | -0.0002*** | 0.16 | 1,354.13 |
| 95% CI | (2.04, 2.31) | (-0.01, 0.02) | (-0.01, 0.01) | (-0.002, 0.001) | (-0.0002, 0.0004) | (-0.17, 0.04) | (-0.06, 0.20) | (-0.02, 0.02) | (-0.002, 0.002) | (-0.01, 0.01) | (-0.001, 0.0001) | (0.0001, 0.0001) | (-0.0002,  -0.0001) |  |  |
| Cohens *d* |  | 0.001 | 0.000 | 0.000 | 0.000 | -0.04 | 0.04 | 0.000 | 0.000 | 0.000 | 0.000 | 0.000 | 0.000 |  |  |
| *p*-value | <.001 | .855 | .847 | .682 | .480 | .215 | .299 | .984 | .664 | .824 | .169 | <.001 | <.001 |  |  |
| Inspiring | -1.18*** | 0.02** | 0.002 | -0.001 | 0.0002 | -0.38*** | -0.01 | -0.01 | 0.0001 | 0.0003 | -0.0003 | 0.0000*** | -0.0001*** | 0.13 | 1,041.88 |
| 95% CI | (-1.30, -1.07) | (0.01, 0.03) | (-0.003, 0.01) | (-0.002, 0.001) | (-0.0000, 0.0005) | (-0.48, -0.29) | (-0.12, 0.10) | (-0.02, 0.01) | (-0.002, 0.002) | (-0.01, 0.01) | (-0.001, 0.0001) | (0.0000, 0.0000) | (-0.0001,  -0.0001) |  |  |
| Cohens *d* |  | 0.01 | 0.001 | 0.000 | 0.000 | -0.21 | -0.01 | -0.004 | 0.000 | 0.000 | 0.000 | 0.000 | 0.000 |  |  |
| *p*-value | <.001 | .002 | .369 | .489 | .114 | <.001 | .846 | .418 | .879 | .927 | .120 | <.001 | <.001 |  |  |
| Beautiful | -2.18*** | -0.01 | -0.003 | -0.0003 | 0.0004* | -0.33*** | -0.31*** | 0.01 | 0.001 | 0.003 | -0.0002 | 0.00 | -0.0001*** | 0.11 | 1,820.97 |
| 95% CI | (-2.32, -2.04) | (-0.02, 0.01) | (-0.01, 0.003) | (-0.002, 0.002) | (0.0001, 0.001) | (-0.46, -0.21) | (-0.45, -0.17) | (-0.01, 0.03) | (-0.002, 0.003) | (-0.01, 0.01) | (-0.001, 0.0002) | (-0.0000, 0.0000) | (-0.0001,  -0.0000) |  |  |
| Cohens *d* |  | -0.003 | -0.002 | 0.000 | 0.000 | -0.18 | -0.17 | 0.004 | 0.000 | 0.002 | 0.000 | 0.000 | 0.000 |  |  |
| *p*-value | <.001 | .488 | .330 | .767 | .022 | <.001 | <.001 | .483 | .582 | .472 | .351 | .192 | .001 |  |  |
| Ingenious | -3.02*** | -0.03*** | -0.01 | 0.001 | -0.0001 | 0.16** | 0.32*** | 0.01 | -0.001 | -0.001 | 0.0001 | -0.00 | 0.00 | 0.12 | 2,005.43 |
| 95% CI | (-3.17, -2.88) | (-0.05, -0.02) | (-0.01, 0.002) | (-0.001, 0.002) | (-0.0005, 0.0002) | (0.05, 0.26) | (0.18, 0.46) | (-0.01, 0.03) | (-0.003, 0.002) | (-0.01, 0.01) | (-0.0004, 0.001) | (-0.0000, 0.0000) | (-0.0000, 0.0001) |  |  |
| Cohens *d* |  | -0.02 | -0.003 | 0.000 | 0.000 | 0.09 | 0.18 | 0.003 | 0.000 | 0.000 | 0.000 | 0.000 | 0.000 |  |  |
| *p*-value | <.001 | <.001 | .145 | .483 | .548 | .005 | <.001 | .524 | .619 | .840 | .695 | .114 | .059 |  |  |
| Courageous | -2.19*** | 0.02** | -0.003 | 0.0002 | 0.0003* | -0.43*** | -0.24** | -0.005 | -0.001 | -0.0001 | -0.0002 | 0.00 | -0.0001*** | 0.15 | 1,862.32 |
| 95% CI | (-2.33, -2.05) | (0.01, 0.04) | (-0.01, 0.003) | (-0.002, 0.002) | (0.0000, 0.001) | (-0.55, -0.30) | (-0.38, -0.10) | (-0.03, 0.02) | (-0.003, 0.001) | (-0.01, 0.01) | (-0.001, 0.0002) | (-0.0000, 0.0000) | (-0.0001,  -0.0000) |  |  |
| Cohens *d* |  | 0.01 | -0.002 | 0.000 | 0.000 | -0.24 | -0.13 | -0.003 | 0.000 | 0.000 | 0.000 | 0.000 | 0.000 |  |  |
| *p*-value | <.001 | .004 | .316 | .846 | .043 | <.001 | .002 | .634 | .520 | .980 | .383 | .164 | <.001 |  |  |
| Jaw-dropping | -3.30*** | 0.0005 | -0.0002 | -0.0002 | 0.00 | 0.07 | 0.15* | -0.01 | -0.0002 | -0.005 | -0.00 | 0.0000* | 0.0001*** | 0.06 | 2,271.17 |
| 95% CI | (-3.44, -3.15) | (-0.02, 0.02) | (-0.01, 0.01) | (-0.002, 0.002) | (-0.0003, 0.0004) | (-0.04, 0.18) | (0.01, 0.29) | (-0.03, 0.01) | (-0.002, 0.002) | (-0.01, 0.003) | (-0.0004, 0.0004) | (0.0000, 0.0000) | (0.0000, 0.0001) |  |  |
| Cohens *d* |  | 0.000 | 0.000 | 0.000 | 0.000 | 0.04 | 0.08 | -0.01 | 0.000 | -0.003 | 0.000 | 0.000 | 0.000 |  |  |
| *p*-value | <.001 | .956 | .961 | .814 | .900 | .227 | .037 | .339 | .836 | .243 | .914 | .037 | <.001 |  |  |
| Fascinating | -2.07*** | -0.02*** | 0.003 | -0.0011 | -0.0001 | 0.38*** | 0.11* | 0.002 | 0.001 | -0.003 | -0.0000 | 0.00 | 0.00 | 0.13 | 1,489.05 |
| 95% CI | (-2.18, -1.96) | (-0.03, -0.01) | (-0.002, 0.01) | (-0.003, 0.0001) | (-0.0003, 0.0002) | (0.30, 0.46) | (0.01, 0.22) | (-0.01, 0.02) | (-0.001, 0.002) | (-0.01, 0.003) | (-0.0004, 0.0003) | (-0.0000, 0.0000) | (-0.0000, 0.0000) |  |  |
| Cohens *d* |  | -0.01 | 0.002 | -0.001 | 0.000 | 0.21 | 0.06 | 0.001 | 0.000 | -0.002 | 0.000 | 0.000 | 0.000 |  |  |
| *p*-value | <.001 | .001 | .275 | .069 | .555 | <.001 | .035 | .794 | .437 | .277 | .866 | .801 | .083 |  |  |

| **Table K (continued)** | | | | | | | | | | | | | | | |
| --- | --- | --- | --- | --- | --- | --- | --- | --- | --- | --- | --- | --- | --- | --- | --- |
| Dependent variables | Intercept | Age-linked language style | Speaker’s age | Age-linked language style (squared) | Speaker’s age (squared) | academic status (academic) | speaker's gender (male) | speaker's gender (male)× Age-linked language style | speaker's gender (male)× Age-linked language style (squared) | speaker's gender (male)× Speaker’s age | speaker's gender (male)× Speaker’s age (squared) | Total ratings | Time online | *R*^2^ | Log Likelihood |
| Informative | -1.54*** | 0.01 | -0.002 | -0.0001 | -0.001** | 0.61*** | -0.01 | -0.003 | 0.0001 | 0.01 | 0.0003 | -0.0000*** | -0.0001*** | 0.14 | 1,146.53 |
| 95% CI | (-1.66, -1.41) | (-0.005, 0.02) | (-0.01, 0.004) | (-0.002, 0.002) | (-0.001, -0.0002) | (0.52, 0.71) | (-0.14, 0.11) | (-0.02, 0.01) | (-0.002, 0.002) | (-0.002, 0.01) | (-0.0001, 0.001) | (-0.0000, -0.0000) | (-0.0001,  -0.0000) |  |  |
| Cohens *d* |  | 0.01 | -0.001 | 0.000 | 0.000 | 0.34 | -0.01 | -0.002 | 0.000 | 0.003 | 0.000 | 0.000 | 0.000 |  |  |
| *p*-value | <.001 | .191 | .516 | .943 | .004 | <.001 | .852 | .753 | .901 | .154 | .108 | <.001 | <.001 |  |  |
| Funny | -3.10*** | -0.03*** | 0.001 | -0.0001 | 0.0003 | -0.11 | 0.03 | 0.01 | 0.001 | -0.001 | -0.0002 | 0.0000** | 0.0001** | 0.07 | 2,302.21 |
| 95% CI | (-3.28, -2.92) | (-0.05, -0.01) | (-0.01, 0.01) | (-0.002, 0.002) | (-0.0001, 0.001) | (-0.25, 0.03) | (-0.13, 0.20) | (-0.01, 0.04) | (-0.002, 0.003) | (-0.01, 0.01) | (-0.001, 0.0003) | (0.0000, 0.0000) | (0.0000, 0.0001) |  |  |
| Cohens *d* |  | -0.02 | 0.000 | 0.000 | 0.000 | -0.06 | 0.02 | 0.01 | 0.000 | 0.000 | 0.000 | 0.000 | 0.000 |  |  |
| *p*-value | <.001 | .001 | .864 | .942 | .208 | .125 | .125 | .277 | .683 | .881 | .536 | .002 | .009 |  |  |
| Persuasive | -2.30*** | 0.03*** | 0.004 | 0.0001 | -0.0002 | 0.17** | 0.08 | -0.01 | -0.001 | 0.004 | -0.0001 | 0.00 | -0.0000* | 0.08 | 1,642.23 |
| 95% CI | (-2.43, -2.16) | (0.02, 0.05) | (-0.003, 0.01) | (-0.002, 0.002) | (-0.001, 0.0001) | (0.06, 0.27) | (-0.05, 0.22) | (-0.03, 0.01) | (-0.003, 0.001) | (-0.004, 0.01) | (-0.001, 0.0004) | (-0.0000, 0.0000) | (-0.0001,  -0.0000) |  |  |
| Cohens *d* |  | 0.02 | 0.002 | 0.000 | 0.000 | 0.09 | 0.05 | -0.003 | 0.000 | 0.002 | 0.000 | 0.000 | 0.000 |  |  |
| *p*-value | <.001 | <.001 | .265 | .916 | .212 | .002 | .002 | .524 | .507 | .356 | .729 | .062 | .017 |  |  |
| *Note.* *N* = 1,095, CI = Confidence interval of estimates. Speaker’s age and age-linked language style were mean-centered prior to model inclusion. All ratings represent percentages of total ratings received. Positive ratings = aggregated score of all positive ratings.  *p<0.05;**p<0.01; ***p<0.001. | | | | | | | | | | | | | | | |

| **Table L**  *Research Question 2, Negative TED Talk Ratings by Age and Age-Linked Language Style: Beta Regression Results* | | | | | | | | | | | | | | | |
| --- | --- | --- | --- | --- | --- | --- | --- | --- | --- | --- | --- | --- | --- | --- | --- |
| Dependent variables | Intercept | Age-linked language style | Speaker’s age | Age-linked language style (squared) | Speaker’s age (squared) | academic status (academic) | speaker's gender (male) | speaker's gender (male)× Age-linked language style | speaker's gender (male)× Age-linked language style (squared) | speaker's gender (male)× Speaker’s age | speaker's gender (male)× Speaker’s age (sqaured) | Total ratings | Time online | *R*^2^ | Log Likelihood |
| Negative ratings | -2.70*** | 0.002 | 0.003 | 0.0003 | -0.0002 | 0.05 | -0.08 | -0.001 | -0.001 | -0.0002 | 0.0003 | -0.0001*** | 0.0002*** | 0.17 | 1,755.28 |
| 95% CI | (-2.85, -2.56) | (-0.01, 0.02) | (-0.003, 0.01) | (-0.001, 0.002) | (-0.001, 0.0001) | (-0.06, 0.16) | (-0.21, 0.06) | (-0.02, 0.02) | (-0.003, 0.001) | (-0.01, 0.01) | (-0.0001, 0.001) | (-0.0001, -0.0000) | (0.0001, 0.0002) |  |  |
| Cohen's *d* |  | 0.001 | 0.002 | 0.000 | 0.000 | 0.03 | -0.04 | -0.001 | 0.000 | 0.000 | 0.000 | 0.000 | 0.000 |  |  |
| *p*-value | <.001 | .790 | .345 | .700 | .229 | .342 | .260 | .925 | .552 | .955 | .104 | <.001 | <.001 |  |  |
| Obnoxious | -4.34*** | 0.002 | 0.0004 | 0.0004 | -0.0003 | -0.08 | -0.14* | -0.001 | -0.001 | -0.002 | 0.0003 | -0.0000*** | 0.0001*** | 0.10 | 3,542.41 |
| 95% CI | (-4.49, -4.20) | (-0.01, 0.02) | (-0.01, 0.01) | (-0.001, 0.002) | (-0.001, 0.0001) | (-0.19, 0.04) | (-0.28, -0.002) | (-0.02, 0.02) | (-0.003, 0.001) | (-0.01, 0.01) | (-0.0001, 0.001) | (-0.0000, -0.0000) | (0.0001, 0.0002) |  |  |
| Cohen's *d* |  | 0.001 | 0.000 | 0.000 | 0.000 | -0.04 | -0.08 | -0.001 | 0.000 | -0.001 | 0.000 | 0.000 | 0.000 |  |  |
| *p*-value | <.001 | .837 | .899 | .693 | .113 | .200 | .047 | .886 | .518 | .583 | .167 | <.001 | <.001 |  |  |
| Longwinded | -4.15*** | 0.002 | 0.01* | -0.0001 | -0.0004* | 0.07 | -0.11 | 0.003 | -0.0003 | 0.001 | 0.0004* | -0.0001*** | 0.0002*** | 0.22 | 3,171.06 |
| 95% CI | (-4.29, -4.02) | (-0.01, 0.02) | (0.001, 0.01) | (-0.002, 0.002) | (-0.001, -0.0000) | (-0.04, 0.17) | (-0.24, 0.02) | (-0.02, 0.02) | (-0.002, 0.002) | (-0.01, 0.01) | (0.0000, 0.001) | (-0.0001, -0.0000) | (0.0002, 0.0003) |  |  |
| Cohen's *d* |  | 0.001 | 0.004 | 0.000 | 0.000 | 0.04 | -0.06 | 0.002 | 0.000 | 0.001 | 0.000 | 0.000 | 0.000 |  |  |
| *p*-value | <.001 | .752 | .030 | .918 | .032 | .209 | .088 | .749 | .771 | .765 | .047 | <.001 | <.001 |  |  |
| Unconvincing | -3.59*** | 0.004 | 0.00 | 0.001 | -0.0003 | 0.03 | -0.08 | -0.0002 | -0.001 | -0.0004 | 0.0005* | -0.0000*** | 0.0001*** | 0.09 | 2,756.67 |
| 95% CI | (-3.73, -3.44) | (-0.01, 0.02) | (-0.01, 0.01) | (-0.001, 0.002) | (-0.001, 0.0000) | (-0.08, 0.15) | (-0.23, 0.06) | (-0.02, 0.02) | (-0.003, 0.001) | (-0.01, 0.01) | (0.0000, 0.001) | (-0.0000, -0.0000) | (0.0001, 0.0001) |  |  |
| Cohen's *d* |  | 0.002 | 0.000 | 0.000 | 0.000 | 0.02 | -0.05 | 0.000 | 0.000 | 0.000 | 0.000 | 0.000 | 0.000 |  |  |
| *p*-value | <.001 | .595 | .993 | .467 | .067 | .591 | .252 | .986 | .475 | .930 | .034 | <.001 | <.001 |  |  |
| Confusing | -4.63*** | -0.01 | 0.001 | 0.001 | 0.00 | 0.18** | 0.07 | 0.001 | -0.001 | 0.001 | -0.0001 | -0.0000*** | 0.0001*** | 0.10 | 3,698.35 |
| 95% CI | (-4.78, -4.49) | (-0.02, 0.01) | (-0.01, 0.01) | (-0.001, 0.003) | (-0.0003, 0.0003) | (0.07, 0.29) | (-0.06, 0.21) | (-0.02, 0.02) | (-0.003, 0.001) | (-0.01, 0.01) | (-0.001, 0.0003) | (-0.0000, -0.0000) | (0.0001, 0.0001) |  |  |
| Cohen's *d* |  | -0.003 | 0.000 | 0.001 | 0.000 | 0.10 | 0.04 | 0.000 | -0.001 | 0.001 | 0.000 | 0.000 | 0.000 |  |  |
| *p*-value | <.001 | .484 | .786 | .235 | .951 | .002 | .300 | .948 | .225 | .777 | .662 | <.001 | <.001 |  |  |
| OK_1_ | -3.18*** | -0.01 | -0.005 | 0.001 | -0.0001 | 0.09* | -0.06 | 0.003 | -0.0003 | 0.003 | 0.0002 | -0.0001*** | 0.0001*** | 0.11 | 2,564.00 |
| 95% CI | (-3.29, -3.07) | (-0.02, 0.005) | (-0.01, 0.0002) | (-0.001, 0.002) | (-0.0004, 0.0002) | (0.003, 0.18) | (-0.17, 0.05) | (-0.01, 0.02) | (-0.002, 0.001) | (-0.003, 0.01) | (-0.0001, 0.001) | (-0.0001, -0.0001) | (0.0001, 0.0001) |  |  |
| Cohen's *d* |  | -0.004 | -0.003 | 0.000 | 0.000 | 0.05 | -0.03 | 0.002 | 0.000 | 0.002 | 0.000 | 0.000 | 0.000 |  |  |
| *p*-value | <.001 | .247 | .059 | .418 | .507 | .042 | .281 | .713 | .746 | .295 | .223 | <.001 | <.001 |  |  |
| *Note.* *N* = 1,095, CI = Confidence interval of estimates. Speaker’s age and age-linked language style were mean-centered prior to model inclusion. All ratings represent percentages of total ratings received. Negative ratings = aggregated score of all negative ratings.  _1_The rating “ok” was not part of the aggregated score of “negative ratings”.  *p<0.05;**p<0.01; ***p<0.001. | | | | | | | | | | | | | | | |

**Figure B**


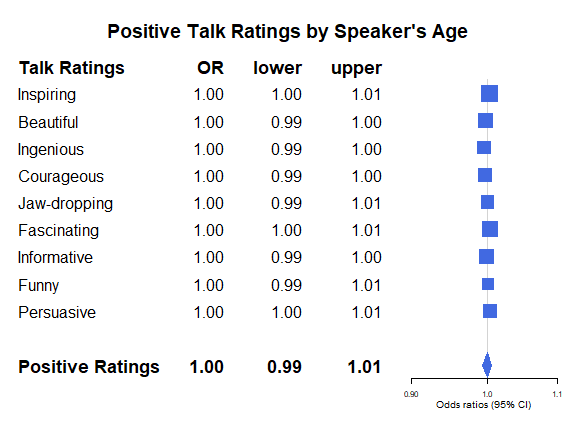


Note. Depicted are exponentiated regression coefficients (OR: Odds ratios) and 95% confidence intervals (lower: lower bound, upper: upper bound) from beta regressions accounting for control variables (age-linked language score linear and squared, gender, academic status, time online, number of ratings). OR > 1 indicate an increased likelihood for talks given by older speakers to receive the rating type. Positive talk ratings (boldface) refers to the aggregated score of all positive ratings

**Figure C**

***
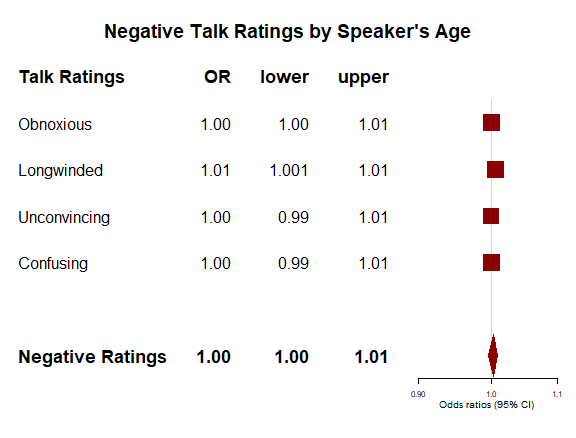
***

Note. Depicted are exponentiated regression coefficients (OR: Odds ratios) and 95% confidence intervals (lower: lower bound, upper: upper bound) from beta regressions accounting for control variables (age-linked language score linear and squared, gender, academic status, time online, number of ratings). OR > 1 indicate an increased likelihood for talks given by older speakers to receive the rating type. Negative talk ratings (boldface) refers to the aggregated score of all negative ratings

Quality of Talk Impact, Average Marginal Effects: Positive and Negative Rating Types by Age and Age-Linked Language (RQ2) (Tables M-N)

Average marginal effects (AME) represent the average model coefficients. As an example, TED Talks, on average, received 19.35% “inspiring” ratings (Table 2). The AME of age-linked language style on “inspiring” was 0.003 (see Table M). A one unit (year) increase in age-linked language style thus corresponded to an 0.3% increase in “inspiring” ratings when all other model predictors were held constant.

| **Table M**  *Research Question 2, Positive TED Talk Ratings by Age and Age-Linked Language Style: Average Marginal Effects From Beta Regressions* | | | | | | |
| --- | --- | --- | --- | --- | --- | --- |
| **Dependent Variable** | Predictor | AME | SE | Lower  CI | Upper  CI | *p* |
| **Positive ratings** |  |  |  |  |  |  |
|  | academic status (academic) | -0.007 | 0.006 | -0.019 | 0.004 | .221 |
|  | Speaker’s age | 0.000 | 0.000 | -0.001 | 0.000 | .540 |
|  | Speaker’s age (squared) | 0.000 | 0.000 | 0.000 | 0.000 | .418 |
|  | speaker's gender (male) | 0.004 | 0.005 | -0.007 | 0.014 | .499 |
|  | Time online | 0.000 | 0.000 | 0.000 | 0.000 | <.001 |
|  | Total ratings | 0.000 | 0.000 | 0.000 | 0.000 | <.001 |
|  | Age-linked language style | 0.000 | 0.001 | -0.001 | 0.001 | .729 |
|  | Age-linked language style (squared) | 0.000 | 0.000 | 0.000 | 0.000 | .928 |
| **Inspiring** |  |  |  |  |  |  |
|  | academic status (academic) | -0.055 | 0.007 | -0.069 | -0.042 | <.001 |
|  | Speaker’s age | 0.000 | 0.000 | 0.000 | 0.001 | .136 |
|  | Speaker’s age (squared) | 0.000 | 0.000 | 0.000 | 0.000 | .761 |
|  | speaker's gender (male) | -0.009 | 0.007 | -0.022 | 0.004 | .196 |
|  | Time online | 0.000 | 0.000 | 0.000 | 0.000 | <.001 |
|  | Total ratings | 0.000 | 0.000 | 0.000 | 0.000 | <.001 |
|  | Age-linked language style | 0.003 | 0.001 | 0.002 | 0.004 | <.001 |
|  | Age-linked language style (squared) | 0.000 | 0.000 | 0.000 | 0.000 | .284 |
| **Beautiful** |  |  |  |  |  |  |
|  | academic status (academic) | -0.021 | 0.004 | -0.028 | -0.014 | <.001 |
|  | Speaker’s age | 0.000 | 0.000 | 0.000 | 0.000 | .560 |
|  | Speaker’s age (squared) | 0.000 | 0.000 | 0.000 | 0.000 | .028 |
|  | speaker's gender (male) | -0.023 | 0.004 | -0.031 | -0.016 | <.001 |
|  | Time online | 0.000 | 0.000 | 0.000 | 0.000 | .001 |
|  | Total ratings | 0.000 | 0.000 | 0.000 | 0.000 | .192 |
|  | Age-linked language style | 0.000 | 0.000 | -0.001 | 0.001 | .799 |
|  | Age-linked language style (squared) | 0.000 | 0.000 | 0.000 | 0.000 | .853 |
| **Ingenious** |  |  |  |  |  |  |
|  | academic status (academic) | 0.010 | 0.004 | 0.003 | 0.017 | .007 |
|  | Speaker’s age | 0.000 | 0.000 | -0.001 | 0.000 | .006 |
|  | Speaker’s age (squared) | 0.000 | 0.000 | 0.000 | 0.000 | .689 |
|  | speaker's gender (male) | 0.018 | 0.003 | 0.012 | 0.023 | <.001 |
|  | Time online | 0.000 | 0.000 | 0.000 | 0.000 | .059 |
|  | Total ratings | 0.000 | 0.000 | 0.000 | 0.000 | .114 |
|  | Age-linked language style | -0.002 | 0.000 | -0.002 | -0.001 | <.001 |
|  | Age-linked language style (squared) | 0.000 | 0.000 | 0.000 | 0.000 | .606 |

| **Table M (continued)** | | | | | | |
| --- | --- | --- | --- | --- | --- | --- |
| **Dependent Variable** | Predictor | AME | SE | Lower  CI | Upper  CI | *p* |
| **Courageous** |  |  |  |  |  |  |
|  | academic status (academic) | -0.025 | 0.004 | -0.032 | -0.018 | <.001 |
|  | Speaker’s age | 0.000 | 0.000 | -0.001 | 0.000 | .127 |
|  | Speaker’s age (squared) | 0.000 | 0.000 | 0.000 | 0.000 | .060 |
|  | speaker's gender (male) | -0.020 | 0.004 | -0.028 | -0.013 | <.001 |
|  | Time online | 0.000 | 0.000 | 0.000 | 0.000 | <.001 |
|  | Total ratings | 0.000 | 0.000 | 0.000 | 0.000 | .164 |
|  | Age-linked language style | 0.001 | 0.000 | 0.001 | 0.002 | <.001 |
|  | Age-linked language style (squared) | 0.000 | 0.000 | 0.000 | 0.000 | .631 |
| **Jaw-dropping** |  |  |  |  |  |  |
|  | academic status (academic) | 0.003 | 0.003 | -0.002 | 0.008 | .235 |
|  | Speaker’s age | 0.000 | 0.000 | 0.000 | 0.000 | .079 |
|  | Speaker’s age (squared) | 0.000 | 0.000 | 0.000 | 0.000 | .966 |
|  | speaker's gender (male) | 0.006 | 0.002 | 0.002 | 0.011 | .006 |
|  | Time online | 0.000 | 0.000 | 0.000 | 0.000 | <.001 |
|  | Total ratings | 0.000 | 0.000 | 0.000 | 0.000 | .036 |
|  | Age-linked language style | 0.000 | 0.000 | -0.001 | 0.000 | .170 |
|  | Age-linked language style (squared) | 0.000 | 0.000 | 0.000 | 0.000 | .440 |
| **Fascinating** |  |  |  |  |  |  |
|  | academic status (academic) | 0.046 | 0.005 | 0.036 | 0.057 | <.001 |
|  | Speaker’s age | 0.000 | 0.000 | 0.000 | 0.000 | .845 |
|  | Speaker’s age (squared) | 0.000 | 0.000 | 0.000 | 0.000 | .232 |
|  | speaker's gender (male) | 0.014 | 0.004 | 0.005 | 0.023 | .001 |
|  | Time online | 0.000 | 0.000 | 0.000 | 0.000 | .083 |
|  | Total ratings | 0.000 | 0.000 | 0.000 | 0.000 | .800 |
|  | Age-linked language style | -0.002 | 0.000 | -0.003 | -0.001 | <.001 |
|  | Age-linked language style (squared) | 0.000 | 0.000 | 0.000 | 0.000 | .027 |
| **Informative** |  |  |  |  |  |  |
|  | academic status (academic) | 0.092 | 0.008 | 0.076 | 0.108 | <.001 |
|  | Speaker’s age | 0.000 | 0.000 | 0.000 | 0.001 | .352 |
|  | Speaker’s age (squared) | 0.000 | 0.000 | 0.000 | 0.000 | .008 |
|  | speaker's gender (male) | 0.006 | 0.006 | -0.006 | 0.018 | .347 |
|  | Time online | 0.000 | 0.000 | 0.000 | 0.000 | <.001 |
|  | Total ratings | 0.000 | 0.000 | 0.000 | 0.000 | <.001 |
|  | Age-linked language style | 0.001 | 0.001 | 0.000 | 0.002 | .069 |
|  | Age-linked language style (squared) | 0.000 | 0.000 | 0.000 | 0.000 | .956 |

| **Table M** **(continued)** | | | | | | |
| --- | --- | --- | --- | --- | --- | --- |
| **Dependent variable** | Predictor | AME | SE | Lower CI | Upper CI | *p* |
| **Funny** |  |  |  |  |  |  |
|  | academic status (academic) | -0.005 | 0.003 | -0.012 | 0.001 | .114 |
|  | Speaker’s age | 0.000 | 0.000 | 0.000 | 0.000 | .953 |
|  | Speaker’s age (squared) | 0.000 | 0.000 | 0.000 | 0.000 | .287 |
|  | speaker's gender (male) | 0.001 | 0.003 | -0.006 | 0.007 | .848 |
|  | Time online | 0.000 | 0.000 | 0.000 | 0.000 | .009 |
|  | Total ratings | 0.000 | 0.000 | 0.000 | 0.000 | .001 |
|  | Age-linked language style | -0.001 | 0.000 | -0.002 | -0.001 | <.001 |
|  | Age-linked language style (squared) | 0.000 | 0.000 | 0.000 | 0.000 | .620 |
| **Persuasive** |  |  |  |  |  |  |
|  | academic status (academic) | 0.014 | 0.005 | 0.005 | 0.024 | .003 |
|  | Speaker’s age | 0.001 | 0.000 | 0.000 | 0.001 | .003 |
|  | Speaker’s age (squared) | 0.000 | 0.000 | 0.000 | 0.000 | .015 |
|  | speaker's gender (male) | 0.004 | 0.004 | -0.004 | 0.012 | .306 |
|  | Time online | 0.000 | 0.000 | 0.000 | 0.000 | .017 |
|  | Total ratings | 0.000 | 0.000 | 0.000 | 0.000 | .061 |
|  | Age-linked language style | 0.002 | 0.000 | 0.002 | 0.003 | <.001 |
|  | Age-linked language style (squared) | 0.000 | 0.000 | 0.000 | 0.000 | .422 |
| *Note.* AME = Average marginal effects (type = response; computed with the R-package “margins”; Leeper, 2018), SE = standard error, CI = 95% Confidence Interval. Speaker’s age and age-linked language style were mean-centered prior to model inclusion. All ratings represent percentages of total ratings received. Positive ratings = aggregated score of all positive ratings. The interaction effects of speaker's gender and speakers' age, age-linked language style, speaker's age (squared) and age-linked language style (squared) were additional predictors in the models, but their AMEs are not presented in this table due to space. | | | | | | |

| **Table N**  *Research Questions 2, Negative TED Talk Ratings by Age and Age-Linked Language Style: Average Marginal Effects From Beta Regressions* | | | | | | |
| --- | --- | --- | --- | --- | --- | --- |
| **Dependent Variable** | Predictor | AME | SE | Lower CI | Upper CI | *p* |
| **Negative ratings** |  |  |  |  |  |  |
|  | academic status (academic) | 0.004 | 0.004 | -0.004 | 0.013 | .348 |
|  | Speaker’s age | 0.000 | 0.000 | 0.000 | 0.001 | .162 |
|  | Speaker’s age (squared) | 0.000 | 0.000 | 0.000 | 0.000 | .701 |
|  | speaker's gender (male) | -0.003 | 0.004 | -0.011 | 0.005 | .475 |
|  | Time online | 0.000 | 0.000 | 0.000 | 0.000 | <.001 |
|  | Total ratings | 0.000 | 0.000 | 0.000 | 0.000 | <.001 |
|  | Age-linked language style | 0.000 | 0.000 | -0.001 | 0.001 | .747 |
|  | Age-linked language style (squared) | 0.000 | 0.000 | 0.000 | 0.000 | .840 |
| **Obnoxious** |  |  |  |  |  |  |
|  | academic status (academic) | -0.001 | 0.001 | -0.003 | 0.001 | .189 |
|  | Speaker’s age | 0.000 | 0.000 | 0.000 | 0.000 | .587 |
|  | Speaker’s age (squared) | 0.000 | 0.000 | 0.000 | 0.000 | .537 |
|  | speaker's gender (male) | -0.002 | 0.001 | -0.003 | 0.000 | .041 |
|  | Time online | 0.000 | 0.000 | 0.000 | 0.000 | <.001 |
|  | Total ratings | 0.000 | 0.000 | 0.000 | 0.000 | <.001 |
|  | Age-linked language style | 0.000 | 0.000 | 0.000 | 0.000 | .884 |
|  | Age-linked language style (squared) | 0.000 | 0.000 | 0.000 | 0.000 | .813 |
| **Longwinded** |  |  |  |  |  |  |
|  | academic status (academic) | 0.002 | 0.001 | -0.001 | 0.004 | .218 |
|  | Speaker’s age | 0.000 | 0.000 | 0.000 | 0.000 | <.001 |
|  | Speaker’s age (squared) | 0.000 | 0.000 | 0.000 | 0.000 | .555 |
|  | speaker's gender (male) | -0.001 | 0.001 | -0.003 | 0.001 | .337 |
|  | Time online | 0.000 | 0.000 | 0.000 | 0.000 | <.001 |
|  | Total ratings | 0.000 | 0.000 | 0.000 | 0.000 | <.001 |
|  | Age-linked language style | 0.000 | 0.000 | 0.000 | 0.000 | .301 |
|  | Age-linked language style (squared) | 0.000 | 0.000 | 0.000 | 0.000 | .524 |
| **Unconvincing** |  |  |  |  |  |  |
|  | academic status (academic) | 0.001 | 0.002 | -0.003 | 0.005 | .593 |
|  | Speaker’s age | 0.000 | 0.000 | 0.000 | 0.000 | .915 |
|  | Speaker’s age (squared) | 0.000 | 0.000 | 0.000 | 0.000 | .993 |
|  | speaker's gender (male) | -0.001 | 0.002 | -0.004 | 0.002 | .623 |
|  | Time online | 0.000 | 0.000 | 0.000 | 0.000 | <.001 |
|  | Total ratings | 0.000 | 0.000 | 0.000 | 0.000 | <.001 |
|  | Age-linked language style | 0.000 | 0.000 | 0.000 | 0.000 | .372 |
|  | Age-linked language style (squared) | 0.000 | 0.000 | 0.000 | 0.000 | .802 |

| **Table N (continued)** | | | | | | |
| --- | --- | --- | --- | --- | --- | --- |
| **Dependent Variable** | Predictor | AME | SE | Lower CI | Upper CI | *p* |
| **Confusing** |  |  |  |  |  |  |
|  | academic status (academic) | 0.002 | 0.001 | 0.001 | 0.004 | .003 |
|  | Speaker’s age | 0.000 | 0.000 | 0.000 | 0.000 | .413 |
|  | Speaker’s age (squared) | 0.000 | 0.000 | 0.000 | 0.000 | .606 |
|  | speaker's gender (male) | 0.000 | 0.001 | -0.001 | 0.002 | .695 |
|  | Time online | 0.000 | 0.000 | 0.000 | 0.000 | <.001 |
|  | Total ratings | 0.000 | 0.000 | 0.000 | 0.000 | <.001 |
|  | Age-linked language style | 0.000 | 0.000 | 0.000 | 0.000 | .268 |
|  | Age-linked language style (squared) | 0.000 | 0.000 | 0.000 | 0.000 | .786 |
| **Ok_1_** |  |  |  |  |  |  |
|  | academic status (academic) | 0.004 | 0.002 | 0.000 | 0.008 | .047 |
|  | Speaker’s age | 0.000 | 0.000 | 0.000 | 0.000 | .122 |
|  | Speaker’s age (squared) | 0.000 | 0.000 | 0.000 | 0.000 | .524 |
|  | speaker's gender (male) | -0.001 | 0.002 | -0.005 | 0.002 | .414 |
|  | Time online | 0.000 | 0.000 | 0.000 | 0.000 | <.001 |
|  | Total ratings | 0.000 | 0.000 | 0.000 | 0.000 | <.001 |
|  | Age-linked language style | 0.000 | 0.000 | -0.001 | 0.000 | .149 |
|  | Age-linked language style (squared) | 0.000 | 0.000 | 0.000 | 0.000 | .321 |
| *Note.* AME = Average marginal effects (type = response; computed with the R-package “margins”; Leeper, 2018), SE = standard error, CI = 95% Confidence Interval. Speaker’s age and age-linked language style were mean-centered prior to model inclusion. The interaction effects of speaker's gender and speakers' age, age-linked language style, speaker's age (squared) and age-linked language style (squared) were additional predictors in the models, but their AMEs are not presented in this table due to space. All ratings represent percentages of total ratings received. Negative ratings = aggregated score of all negative ratings._1_The rating “ok” was not part of the aggregated score of “negative ratings”. | | | | | | |

Quantity of Talk Impact, Additional Analysis During Peer-Review (Tables O-P)

| **Table O**  *TED Talk Impact (Number of Views) by Gender-Linked and Analytical Language Style: Quantile Regression Results* | | | | | | | | | | | | | | | |
| --- | --- | --- | --- | --- | --- | --- | --- | --- | --- | --- | --- | --- | --- | --- | --- |
|  | Quantile | | | | | | | | | | | | | | |
|  | 10% | | | 25% | | | 50% | | | 75% | | | 90% | | |
|  | *B (SE)* | *t* | *p* | *B (SE)* | *t* | *p* | *B (SE)* | *t* | *p* | *B (SE)* | *t* | *p* | *B (SE)* | *t* | *p* |
| (Intercept) | 1,042,317.36 (78,219.29) | 13.33 | <.001^***^ | 13,09,744.36 (91,977.53) | 14.24 | <.001^***^ | 2,047,035.73 (132,486.17) | 15.45 | <.001^***^ | 3,131,294.75 (372,296.16) | 8.41 | <.001^***^ | 7,248,790.15 (1,255,317.58) | 5.77 | <.001^***^ |
| Academic status (academic) | 50,549.06 (24,262.75) | 2.08 | 0.037^*^ | 10,0332.62 (49,259.27) | 2.04 | 0.042^*^ | 82,258.01 (50,306.88) | 1.64 | 0.102 | -33,157.02 (174,129.87) | -0.19 | 0.849 | 381,456.47 (438,752.80) | 0.87 | 0.385 |
| Speaker's gender (male) | 2,211.02 (36,498.91) | 0.06 | 0.952 | 37,224.00 (35,674.09) | 1.04 | 0.297 | 137,777.26 (50,458.97) | 2.73 | 0.006^**^ | 54,382.17 (149,514.07) | 0.36 | 0.716 | 530,005.90 (476,241.18) | 1.11 | 0.266 |
| Time online | -120.35 (9.58) | -12.56 | <.001^***^ | -135.93 (13.08) | -10.40 | <.001^***^ | -123.03 (18.36) | -6.70 | <.001^***^ | -65.62 (52.73) | -1.24 | 0.214 | -18.24 (154.31) | -0.12 | 0.906 |
| Speaker's age | -708.57 (888.73) | -0.80 | 0.425 | -694.88 (1,244.48) | -0.56 | 0.577 | -3,027.91 (1,978.10) | -1.53 | 0.126 | -4,175.47 (4,938.57) | -0.85 | 0.398 | -18,203.89 (16458.90) | -1.11 | 0.269 |
| Female language | -968.37 (24,074.70) | -0.04 | 0.968 | 30,046.02 (24,336.73) | 1.23 | 0.217 | 58,821.10 (36,842.47) | 1.60 | 0.111 | 167,414.92 (114,105.56) | 1.47 | 0.143 | 305,390.49 (205,110.05) | 1.49 | 0.137 |
| Analytical language | -1,478.06 (812.79) | -1.82 | 0.069 | -2,744.75 (1,019.14) | -2.69 | 0.007^**^ | -8,521.89 (1,457.65) | -5.85 | <.001^***^ | -15,944.15 (4,107.60) | -3.88 | <.001^***^ | -54,809.63 (12,190.60) | -4.50 | <.001^***^ |
| Speaker's gender × female language | 5,606.14 (28,928.87) | 0.19 | 0.846 | -29,987.70 (35,192.83) | -0.85 | 0.394 | -54,759.01 (50,075.44) | -1.09 | 0.274 | -119,007.45 (143,783.46) | -0.83 | 0.408 | 110,205.23 (369,125.36) | 0.30 | 0.765 |
| *Note.* Gender-linked language (“female language”) was z-standardized prior to inclusion in model. Higher values refer to a more female-typical style. Analytical language was measured with the text analysis program LIWC2015 (Pennebaker et al., 2015); see also Pennebaker et al. (2014). Degrees of freedom: 1,095 total; 1,087 residual.  *p<0.05; **p<0.01; ***p<0.001 | | | | | | | | | | | | | | | |

| **Table P**  *TED Talk Impact (Number of Views) by Age-Linked and Analytical Language Style: Quantile Regression Results* | | | | | | | | | | | | | | | |
| --- | --- | --- | --- | --- | --- | --- | --- | --- | --- | --- | --- | --- | --- | --- | --- |
|  | Quantile | | | | | | | | | | | | | | |
|  | 10% | | | 25% | | | 50% | | | 75% | | | 90% | | |
|  | *B (SE)* | *t* | *p* | *B (SE)* | *t* | *p* | *B (SE)* | *t* | *p* | *B (SE)* | *t* | *p* | *B (SE)* | *t* | *p* |
| (Intercept) | 1,031,264.34 (66,762.58) | 15.45 | <.001^***^ | 1,335,984.90 (67,414.41) | 19.82 | <.001^***^ | 2,030,187.13 (116,662.37) | 17.40 | <.001^***^ | 3,337,187.79 (338,467.35) | 9.86 | <.001^***^ | 6,813,066.22 (1,144,040.72) | 5.96 | <.001^***^ |
| Academic status (academic) | 35,710.76 (29,271.85) | 1.22 | 0.223 | 103,717.62 (46,578.14) | 2.23 | 0.026^*^ | 78,064.96 (51,399.19) | 1.52 | 0.129 | -26,148.47 (127,945.73) | -0.20 | 0.838 | 214,064.74 (456,666.94) | 0.47 | 0.639 |
| Speaker's gender (male) | 5,935.54 (43,188.99) | 0.14 | 0.891 | -14,552.34 (42,537.87) | -0.34 | 0.732 | 48,093.22 (70,351.47) | 0.68 | 0.494 | -84,943.28 (149,479.36) | -0.57 | 0.570 | 239,802.56 (516,270.18) | 0.46 | 0.642 |
| Time online | -121.72 (10.62) | -11.46 | <.001^***^ | -135.24 (12.13) | -11.15 | <.001^***^ | -120.46 (18.17) | -6.63 | <.001^***^ | -94.29 (41.04) | -2.30 | 0.022^*^ | -4.56 (126.79) | -0.04 | 0.971 |
| Age-linked language style | 313.02 (6,295.92) | 0.05 | 0.960 | 4,448.52 (3,997.72) | 1.11 | 0.266 | 5,163.10 (9,078.48) | 0.57 | 0.570 | -10,315.27 (14,517.78) | -0.71 | 0.478 | -10,964.47 (98,028.45) | -0.11 | 0.911 |
| Speaker’s age | 572.79 (1,753.36) | 0.33 | 0.744 | -868.27 (1,153.67) | -0.75 | 0.452 | -4,107.15 (2,013.64) | -2.04 | 0.042^*^ | 6,349.79 (5,040.50) | 1.26 | 0.208 | -10,189.63 (24,062.64) | -0.42 | 0.672 |
| Age-linked language style  (squared) | -336.34 (657.85) | -0.51 | 0.609 | -236.07 (614.35) | -0.38 | 0.701 | -1,015.20 (1,035.67) | -0.98 | 0.327 | -64.88 (1,870.73) | -0.03 | 0.972 | 2,692.82 (10,512.92) | 0.26 | 0.798 |
| Speaker’s age (squared) | 110.65 (65.94) | 1.68 | 0.094 | 62.86 (73.88) | 0.85 | 0.395 | -18.12 (74.98) | -0.24 | 0.809 | -569.65 (278.86) | -2.04 | 0.041^*^ | -664.96 (1,945.22) | -0.34 | 0.733 |
| Analytical language | -1,777.19 (786.45) | -2.26 | 0.024^*^ | -3,765.91 (860.88) | -4.37 | <.001^***^ | -10,202.15 (1,397.47) | -7.30 | <.001^***^ | -20,016.99 (3,367.08) | -5.94 | <.001^***^ | -60,317.57 (13,238.07) | -4.56 | <.001^***^ |
| speaker's gender (male)× Speaker’s age | -2,420.92 (2648.61) | -0.91 | 0.361 | -3,079.84 (2,632.99) | -1.17 | 0.242 | 3,061.25 (2,844.02) | 1.08 | 0.282 | -20,846.21 (10,867.84) | -1.92 | 0.055 | -18,130.24 (40,953.02) | -0.44 | 0.658 |
| speaker's gender (male)× Speaker’s age(squared) | -27.29 (142.43) | -0.19 | 0.848 | 218.77 (146.78) | 1.49 | 0.136 | 71.35 (127.18) | 0.56 | 0.575 | 1,131.95 (724.27) | 1.56 | 0.118 | 1,385.13 (2,356.17) | 0.59 | 0.557 |
| speaker's gender (male)× Age-linked language style | 1,285.36 (7,610.13) | 0.17 | 0.866 | -2,689.41 (6,759.71) | -0.40 | 0.691 | -5,114.45 (10,157.64) | -0.50 | 0.615 | 25,870.54 (21,971.90) | 1.18 | 0.239 | 49,397.21 (104,968.12) | 0.47 | 0.638 |
| speaker's gender (male)× Age-linked language style  (squared) | 80.70 (963.17) | 0.08 | 0.933 | 613.82 (963.49) | 0.64 | 0.524 | 2,137.09 (1,128.42) | 1.89 | 0.059 | -81.29 (3,113.13) | -0.03 | 0.979 | -3,107.08 (10,801.80) | -0.29 | 0.774 |
| *Note.* Speaker’s age and age-linked language style were mean-centered prior to model inclusion. Analytical language was measured with the text analysis program LIWC2015 (Pennebaker et al., 2015); see also Pennebaker et al. (2014). Degrees of freedom: 1095 total; 1082 residual  *p<0.05; **p<0.01; ***p<0.001 | | | | | | | | | | | | | | | |

References

Leeper, T. J. (2018). *Margins: Marginal effects for model objects* (Version 0.3.23) [Computer software]. https://cran.r-project.org/web/packages/margins/margins.pdf

Pennebaker, J. W., Boyd, R. L., Jordan, K., & Blackburn, K. (2015). *The Development and Psychometric Properties of LIWC2015.* https://doi.org/10.15781/T29G6Z

Pennebaker, J. W., Chung, C. K., Frazee, J., Lavergne, G. M., & Beaver, D. I. (2014). When small words foretell academic success: The case of college admissions essays. *PloS One*, *9*(12), e115844. https://doi.org/10.1371/journal.pone.0115844

Smithson, M., & Verkuilen, J. (2006). A better lemon squeezer? Maximum-likelihood regression with beta-distributed dependent variables. *Psychological Methods*, *11*(1), 54–71. https://doi.org/10.1037/1082-989X.11.1.54
